# Frailty Transforms Care Continuity‐Mortality Relationships Across Age Groups: Evidence From Taiwan and South Korea

**DOI:** 10.1002/jcsm.70303

**Published:** 2026-05-14

**Authors:** Fei‐Yuan Hsiao, Sunyoung Kim, Lin‐Chieh Meng, Jae‐ryun Lee, Ho‐Min Chen, Chih‐Kuang Liang, Li‐Ning Peng, Hyejin Lee, Liang‐Kung Chen

**Affiliations:** ^1^ Graduate Institute of Clinical Pharmacy, College of Medicine National Taiwan University Taipei Taiwan; ^2^ School of Pharmacy, College of Medicine National Taiwan University Taipei Taiwan; ^3^ Department of Pharmacy National Taiwan University Hospital Taipei Taiwan; ^4^ Department of Family Medicine, Kyung Hee University Medical Center Kyung Hee University College of Medicine Seoul South Korea; ^5^ Center for Digital Health, Medical Science Research Institute Kyung Hee University College of Medicine Seoul South Korea; ^6^ Health Data Research Center National Taiwan University Taipei Taiwan; ^7^ Department of Biostatistics, Epidemiology and Informatics, Perelman School of Medicine University of Pennsylvania Philadelphia USA; ^8^ Department of Family Medicine Seoul National University Bundang Hospital Seongnam South Korea; ^9^ Center for Geriatrics and Gerontology Kaohsiung Veterans General Hospital Kaohsiung Taiwan; ^10^ Center for Healthy Longevity and Aging Sciences National Yang Ming Chiao Tung University Taipei Taiwan; ^11^ Center for Geriatrics and Gerontology Taipei Veterans General Hospital Taipei Taiwan; ^12^ Department of Family Medicine Seoul National University College of Medicine Seoul South Korea; ^13^ Taipei Municipal Gan‐Dau Hospital Taipei Taiwan

**Keywords:** ageing population, care fragmentation, frailty, healthcare utilisation, mortality

## Abstract

**Background:**

Healthcare fragmentation typically predicts poor outcomes, yet its relationship with frailty across different age groups remains unexplored. We examined how frailty modifies the association between care patterns and mortality in middle‐aged and older adults from two rapidly ageing Asian societies.

**Methods:**

We analysed national insurance data from Taiwan (*n* = 370 997) and South Korea (*n* = 392 466) for adults aged ≥ 45 years, categorised as middle‐aged (45–64) and older (≥ 65) groups. Frailty was assessed using a validated multimorbidity frailty index. We measured healthcare utilisation, care fragmentation (Usual Provider of Care [UPC], Bice‐Boxerman Continuity of Care Index [COCI], Sequential Continuity Index [SECON]) and 3‐year mortality. Cox proportional hazards models were used to estimate adjusted hazard ratios(aHRs) between healthcare utilisation as well as care fragmentation and risk of mortailty.

**Results:**

Frailty prevalence increased with age in both countries (Taiwan: 19.2% middle‐aged, 53.8% older adults; South Korea: 24.7%, 63.0%). Healthcare utilisation increased sharply with frailty severity. Among middle‐aged adults, severely frail individuals had substantially more outpatient visits than fit individuals (Taiwan: 56.5 ± 31.0 versus 13.2 ± 10.4; South Korea: 67.1 ± 55.1 versus 13.3 ± 11.7). Hospitalisation rates rose dramatically from 6.1% (fit) to 70.9% (severely frail) in middle‐aged and 6.8% (fit) to 68.6% (severely frail) in older Taiwanese, with similar increases in South Korea (10.8% to 67.5% in middle‐aged and 10.2% to 62.5% in older groups, respectively). Care continuity declined with increasing frailty. In Taiwan, UPC decreased from 0.81 ± 0.18 in fit to 0.69 ± 0.17 in the severely frail among middle‐aged adults, with similar declines in COCI and SECON. Declines were less pronounced in South Korea, where severely frail older adults maintained higher continuity (UPC 0.78 ± 0.16) than their Taiwanese counterparts (0.68 ± 0.17). During 3‐year follow‐up, higher care fragmentation was associated with increased mortality among fit middle‐aged adults (aHR 1.69, 95% CI 1.14–2.50) but showed inverse associations among moderately frail individuals (aHR 0.67, 95% CI 0.31–1.42), consistently across both countries and age groups.

**Conclusion:**

Frailty, not age, fundamentally transforms how care patterns relate to mortality. The fragmentation‐mortality paradox across age groups indicates that frailty‐specific coordination approaches are needed throughout the adult life course. Healthcare systems must implement standardised frailty service frameworks, develop accessible specialised geriatric services and design integrated care models that optimise the quality, efficiency and sustainability of healthcare systems in rapidly ageing societies.

## 1 Introduction

Ageing is characterised by diminished physiological reserve, dysregulated homeostasis, impaired stress responses and disrupted inflammatory pathways that ultimately translate to decreased organ function and development of multimorbidity [1, 2]. These biological alterations, in conjunction with social vulnerability, contribute to complex care needs that collectively eventuate in adverse clinical outcomes among older populations [3, 4]. These converging biological and social challenges are further compounded in countries experiencing rapid population ageing face where socioeconomic infrastructure has insufficient time to adapt to the demographic transition [5, 6, 7]. In this context, how healthcare systems respond to rapid population ageing constitute significant determinants of national healthcare quality, expenditure and clinical outcomes of older individuals.

Although population ageing correlates with increased healthcare utilisation, demographic transition accounts for only 0.5%–0.7% of annual healthcare expenditure growth [8, 9]. The disproportionate healthcare utilisation observed in older adults stems primarily from accumulated multimorbidity, geriatric syndromes and functional disabilities, rather than chronological age per se [10, 11]. Healthcare utilisation patterns of older people are further modulated by access disparities, care coordination failures, provider fragmentation and health literacy limitations [12, 13]. Beyond these clinical and care delivery factors, broader healthcare system drivers, most notably technological innovation including advanced diagnostics and novel therapies, contribute substantially to rising healthcare expenditure in high‐income countries, accounting for an estimated 30%–50% of spending growth [14].

Taiwan and South Korea represent paradigmatic cases of rapidly ageing societies with comparable yet different healthcare system characteristics [15]. Both countries exhibit similar socioeconomic development trajectories, populations with approximately 20% older adults (≥ 65 years), accelerated demographic transitions and analogous life expectancy metrics [16, 17, 18]. At the financing level, their healthcare infrastructures demonstrate structural similarity, with both implementing universal health coverage through single‐payer national health insurance schemes and establishing long‐term care frameworks in recent decades. However, despite these similarities, the organisation and delivery of healthcare differ between the two systems. Although both systems lack robust primary healthcare gatekeeping mechanisms and permit relatively unrestricted provider selection, they differ in the integration between hospital and primary care sectors, the availability and distribution of long‐term care services, care coordination mechanisms and referral patterns. These delivery‐level differences may contribute to care fragmentation and suboptimal resource allocation, potentially among those with frailty or complex care needs [19].

To explore the complex interrelationships between frailty, care fragmentation and health outcomes in rapidly ageing societies, we conducted analysis using longitudinal National Health Insurance datasets from Taiwan and South Korea to examine healthcare utilisation patterns in adults aged ≥ 45 years across distinct geopolitical contexts. Specifically, we sought to (1) quantify how healthcare utilisation and care continuity metrics change across the frailty spectrum; (2) determine whether the association between care fragmentation and mortality differs by frailty status; and (3) assess whether these relationships remain consistent in different age groups across two rapidly ageing societies.

## 2 Methods

### 2.1 Data Source

This cross‐national study utilised population‐based data from Taiwan and South Korea. The Taiwanese cohort was derived from Taiwan's National Health Insurance (NHI) database, a nationwide claims database that covers approximately 99% of the Taiwanese population, totalling around 23.5 million individuals [20]. The NHI database includes detailed patient‐level information from outpatient consultations, emergency department (ED) visits, hospital admissions and pharmacy dispensing records. The database contains extensive data on healthcare utilisation, prescribed medications and diagnostic codes (ICD‐10) for each medical encounter. To ensure confidentiality, all identifiable information within the NHI database was encrypted. The South Korean cohort utilised the South Korea NHI database [21]. This database encompasses a near‐total representation of the South Korean population, with 97% comprising NHI service beneficiaries and the remaining 3% consisting of Medical Aid recipients. Data for both groups are comprehensively integrated within the NHI service database. Similar to the Taiwanese NHI database, this resource provides a comprehensive dataset encompassing healthcare utilisation patterns, diagnostic information and prescription drug details. This study was approved by the Institutional Review Board of National Taiwan University Hospital (201803134RINC) and Seoul National University Bundang Hospital (X‐2406‐904‐901), and the requirement for individual informed consent was waived due to its retrospective study design.

### 2.2 Study Population and Design

The same study protocol was applied across both cohorts. Data were collected to construct a 4‐year panel of claims spanning from 2017 to 2020 for analytical purposes. To reduce computational demands while preserving the representativeness of the national cohort, a random subset of 1 000 000 beneficiaries was selected from both databases in 2017 [11], stratified by age and sex. The study included all individuals aged 45 years or older, who were further categorised into distinct age groups: middle‐aged (45–64 years) and older aged (65 years and above).

To examine the impact of frailty on healthcare utilisation, care fragmentation and mortality, a frailty assessment was performed for each eligible participant in 2017. Frailty was evaluated using the multimorbidity frailty index (mFI), a 38‐item tool based on ICD‐10 codes [22]. The mFI, originally developed and validated using Taiwan's NHI database, has been widely used in previous studies utilising the same database [23, 24, 25], as well as in different healthcare settings [26, 27]. In this study, we additionally applied the mFI to a South Korean cohort to examine the consistency of frailty patterns and their associations with healthcare utilisation and mortality in a different healthcare system. The index follows a binary scoring system, assigning a score of 1 for the presence of a deficit and 0 for its absence. The mFI score is calculated as the ratio of the total number of deficits to the total possible deficits, resulting in a value ranging from 0 to 1. Higher mFI scores indicate greater frailty, whereas lower scores suggest lesser frailty. Participants were categorised into four groups based on their mFI scores [22]: fit, mildly frail, moderately frail and severely frail.

### 2.3 Healthcare Utilisation and Care Fragmentation

Healthcare utilisation and care fragmentation among the study participants were assessed using data from 2017. Measures of healthcare utilisation included the total number of outpatient visits, the number of unique healthcare providers consulted for outpatient care, transitions between different providers for outpatient services, hospital admissions, ED visits and the duration of hospital stays (LOS).

Care fragmentation was assessed using well‐established indices that quantify different dimensions of care continuity [28, 29]. These indices comprised (1) the Usual Provider of Care Index (UPC) [30], which evaluates the proportion of visits concentrated with a single provider; (2) the Bice‐Boxerman Continuity of Care Index (COCI) [31], which represents how evenly care is distributed among multiple providers; and (3) the Sequential Continuity Index (SECON) [28], which measures the consistency of visits to the same provider over consecutive encounters. All indices range from 0 to 1, with higher scores indicating greater continuity of care and lower scores reflecting more fragmented care.

### 2.4 Outcome of Interest

The outcome of interest was all‐cause mortality, determined using the date of death recorded in the National Death Registry in Taiwan and NHI service database in South Korea. Participants were followed from 1 January 2018, until the date of death or the last available date in the database (31 December 2020), whichever occurred first.

### 2.5 Other Variables

Demographic variables included age (categorised as 45–54, 55–64, 65–74, 75–84 or ≥ 85 years) and sex (male/female). Comorbidity was assessed using the Charlson Comorbidity Index (CCI) [32] based on data from the baseline period.

### 2.6 Statistical Analysis

Descriptive analysis was performed to summarise baseline characteristics, healthcare utilisation and care fragmentation by age group and frailty status in 2017. Continuous variables in the text and tables are presented as the mean ± standard deviation, whereas categorical variables are reported as percentages. Cox proportional hazards models were employed to evaluate the association between healthcare utilisation (hospital admissions, ED visits and care fragmentation) as well as care fragmentation with all‐cause mortality, stratified by age group and frailty status while adjusting for age and sex. The adjusted hazard ratio (HR) and 95% confidence interval (CI) were reported. All statistical analyses were conducted using SAS software, version 9.4 (SAS Institute, Cary, NC, USA).

## 3 Results

### 3.1 Demographics of Study Participants

Our cross‐national study included 370 997 participants from Taiwan (Table 1) and 392 466 from South Korea (Table 2), with similar age distributions of approximately 63%–65% middle‐aged (45–64 years) and 35%–37% older adults (≥ 65 years). Frailty prevalence increased dramatically with age in both countries, with 19.2% of middle‐aged Taiwanese showing a certain degree of frailty compared to 53.8% of older adults (*p* < 0.001). South Korea showed a comparable pattern with slightly higher frailty prevalence, particularly in mild and moderate categories (24.7% of middle‐aged and 63.0% of older adults classified as frail).

**TABLE 1:** Baseline characteristics of study participants in Taiwan, stratified by frailty status.

	Total		Fit		Mild frailty		Moderate frailty		Severe frailty	
	*n*	(%)	*n*	(%)	*n*	(%)	*n*	(%)	*n*	(%)
Aged 45–64 years										
Number	232 647		187 945		38 321		5341		1040	
Age, years										
Mean (SD)	54.7	(5.7)	54.2	(5.6)	56.5	(5.4)	57.8	(5.1)	58.4	(5.1)
45–54	113 113	(48.6)	98 217	(52.3)	13 310	(34.7)	1349	(25.3)	237	(22.8)
55–64	119 534	(51.4)	89 728	(47.7)	25 011	(65.3)	3992	(74.7)	803	(77.2)
Sex										
Male	107 271	(46.1)	83 244	(44.3)	20 362	(53.1)	3026	(56.7)	639	(61.4)
Female	125 376	(53.9)	104 701	(55.7)	17 959	(46.9)	2315	(43.3)	401	(38.6)
Comorbidity										
Myocardial infarction	1239	(0.5)	455	(0.2)	555	(1.4)	173	(3.2)	56	(5.4)
Congestive heart failure	2560	(1.1)	454	(0.2)	1310	(3.4)	539	(10.1)	257	(24.7)
Peripheral vascular	1170	(0.5)	628	(0.3)	388	(1.0)	119	(2.2)	35	(3.4)
Cerebrovascular disease	5552	(2.4)	1631	(0.9)	2753	(7.2)	862	(16.1)	306	(29.4)
Dementia	187	(0.1)	104	(0.1)	57	(0.1)	17	(0.3)	9	(0.9)
Chronic pulmonary disease	6826	(2.9)	2730	(1.5)	2896	(7.6)	880	(16.5)	320	(30.8)
Rheumatologic disease	1909	(0.8)	1294	(0.7)	475	(1.2)	119	(2.2)	21	(2.0)
Peptic ulcer disease	11 777	(5.1)	4202	(2.2)	5588	(14.6)	1529	(28.6)	458	(44.0)
Mild liver disease	12 138	(5.2)	8364	(4.5)	3019	(7.9)	605	(11.3)	150	(14.4)
Diabetes	25 947	(11.2)	16 798	(8.9)	7309	(19.1)	1450	(27.1)	390	(37.5)
Diabetes with chronic complication	6291	(2.7)	3414	(1.8)	2079	(5.4)	594	(11.1)	204	(19.6)
Hemiplegia or paraplegia	567	(0.2)	170	(0.1)	245	(0.6)	114	(2.1)	38	(3.7)
Renal disease	4317	(1.9)	1021	(0.5)	2249	(5.9)	761	(14.2)	286	(27.5)
Malignancy, including leukaemia and lymphoma	8781	(3.8)	6367	(3.4)	1945	(5.1)	380	(7.1)	89	(8.6)
Moderate or severe liver disease	392	(0.2)	210	(0.1)	130	(0.3)	34	(0.6)	18	(1.7)
Metastatic solid tumour	1194	(0.5)	823	(0.4)	294	(0.8)	66	(1.2)	11	(1.1)
AIDS	250	(0.1)	204	(0.1)	38	(0.1)	5	(0.1)	3	(0.3)
Charlson Comorbidity Index										
Mean (SD)	0.5	(1.2)	0.4	(1.0)	1.1	(1.5)	2.0	(2.0)	3.3	(2.4)
0	166 771	(71.7)	148 677	(79.1)	17 000	(44.4)	1037	(19.4)	57	(5.5)
1	38 701	(16.6)	24 939	(13.3)	11 899	(31.1)	1678	(31.4)	185	(17.8)
2	10 997	(4.7)	5278	(2.8)	4433	(11.6)	1062	(19.9)	224	(21.5)
3	8988	(3.9)	5790	(3.1)	2433	(6.3)	599	(11.2)	166	(16.0)
4	3384	(1.5)	1610	(0.9)	1255	(3.3)	396	(7.4)	123	(11.8)
5+	3806	(1.6)	1651	(0.9)	1301	(3.4)	569	(10.7)	285	(27.4)
Multimorbidity Frailty Index										
Mean (SD)	0.020	(0.029)	0.009	(0.012)	0.060	(0.012)	0.113	(0.012)	0.175	(0.027)
Aged 65+ years										
Number	138 350		63 877		49 514		17 602		7358	
Age, years										
Mean (SD)	74.4	(7.7)	72.3	(6.8)	75.1	(7.7)	77.6	(7.9)	80.1	(8.0)
65–74	78 484	(56.7)	44 070	(69.0)	25 725	(52.0)	6727	(38.2)	1962	(26.7)
75–84	42 577	(30.8)	15 353	(24.0)	17 050	(34.4)	7152	(40.6)	3022	(41.1)
85+	17 289	(12.5)	4454	(7.0)	6738	(13.6)	3723	(21.2)	2374	(32.3)
Sex										
Male	62 730	(45.3)	27 468	(43.0)	22 629	(45.7)	8665	(49.2)	3968	(53.9)
Female	75 620	(54.7)	36 409	(57.0)	26 884	(54.3)	8937	(50.8)	3390	(46.1)
Comorbidity										
Myocardial infarction	1702	(1.2)	293	(0.5)	644	(1.3)	425	(2.4)	340	(4.6)
Congestive heart failure	6862	(5.0)	424	(0.7)	2395	(4.8)	2085	(11.8)	1958	(26.6)
Peripheral vascular	2387	(1.7)	625	(1.0)	955	(1.9)	514	(2.9)	293	(4.0)
Cerebrovascular disease	14 411	(10.4)	1966	(3.1)	6108	(12.3)	3786	(21.5)	2551	(34.7)
Dementia	2483	(1.8)	541	(0.8)	904	(1.8)	595	(3.4)	443	(6.0)
Chronic pulmonary disease	12 283	(8.9)	1731	(2.7)	4587	(9.3)	3337	(19.0)	2628	(35.7)
Rheumatologic disease	1437	(1.0)	540	(0.8)	523	(1.1)	263	(1.5)	111	(1.5)
Peptic ulcer disease	12 861	(9.3)	1552	(2.4)	5133	(10.4)	3762	(21.4)	2414	(32.8)
Mild liver disease	7928	(5.7)	3100	(4.9)	2970	(6.0)	1256	(7.1)	602	(8.2)
Diabetes	31 316	(22.6)	11 389	(17.8)	12 365	(25.0)	5109	(29.0)	2453	(33.3)
Diabetes with chronic complication	10 611	(7.7)	3213	(5.0)	4187	(8.5)	2057	(11.7)	1154	(15.7)
Hemiplegia or paraplegia	806	(0.6)	92	(0.1)	263	(0.5)	240	(1.4)	211	(2.9)
Renal disease	9711	(7.0)	991	(1.6)	3941	(8.0)	2830	(16.1)	1949	(26.5)
Malignancy, including leukaemia and lymphoma	9933	(7.2)	3614	(5.7)	3674	(7.4)	1778	(10.1)	867	(11.8)
Moderate or severe liver disease	257	(0.2)	75	(0.1)	90	(0.2)	52	(0.3)	40	(0.5)
Metastatic solid tumour	1035	(0.7)	363	(0.6)	398	(0.8)	176	(1.0)	98	(1.3)
AIDS	24	(0.0)	15	(0.0)	Combined into 9					
Charlson Comorbidity Index										
Mean (SD)	1.2	(1.7)	0.7	(1.3)	1.4	(1.7)	2.2	(2.0)	3.2	(2.3)
0	62 753	(45.4)	40 812	(63.9)	18 148	(36.7)	3289	(18.7)	504	(6.8)
1	34 812	(25.2)	13 247	(20.7)	15 325	(31.0)	4968	(28.2)	1272	(17.3)
2	15 924	(11.5)	4013	(6.3)	6919	(14.0)	3423	(19.4)	1569	(21.3)
3	11 360	(8.2)	3459	(5.4)	4297	(8.7)	2338	(13.3)	1266	(17.2)
4	6125	(4.4)	1316	(2.1)	2447	(4.9)	1457	(8.3)	905	(12.3)
5+	7376	(5.3)	1030	(1.6)	2377	(4.8)	2127	(12.1)	1842	(25.0)
Multimorbidity Frailty Index										
Mean (SD)	0.054	(0.049)	0.015	(0.013)	0.063	(0.013)	0.115	(0.013)	0.185	(0.036)

**TABLE 2:** Baseline characteristics of study participants in South Korea, stratified by frailty status.

	Total		Fit		Mild frailty		Moderate frailty		Severe frailty	
	*n*	(%)	*n*	(%)	*n*	(%)	*n*	(%)	*n*	(%)
Aged 45–64 years										
Number	254 331		191 580		51 013		9637		2101	
Age, years										
Mean (SD)	54.5	(5.7)	53.8	(5.6)	56.3	(5.4)	57.7	(5.0)	58.4	(4.8)
45–54	126 010	(49.5)	104 998	(54.8)	18 206	(35.7)	2390	(24.8)	416	(19.8)
55–64	128 321	(50.5)	86 582	(45.2)	32 807	(64.3)	7247	(75.2)	1685	(80.2)
Sex										
Male	119 705	(47.1)	89 575	(46.8)	24 578	(48.2)	4525	(47.0)	1027	(48.9)
Female	134 626	(52.9)	102 005	(53.2)	26 435	(51.8)	5112	(53.0)	1074	(51.1)
Comorbidity										
Myocardial infarction	982	(0.4)	200	(0.1)	550	(1.1)	170	(1.8)	62	(3.0)
Congestive heart failure	1625	(0.6)	181	(0.1)	836	(1.6)	426	(4.4)	182	(8.7)
Peripheral vascular	4971	(2.0)	2468	(1.3)	1816	(3.6)	505	(5.2)	182	(8.7)
Cerebrovascular disease	5709	(2.2)	1287	(0.7)	2872	(5.6)	1109	(11.5)	441	(21.0)
Dementia	640	(0.3)	248	(0.1)	253	(0.5)	92	(1.0)	47	(2.2)
Chronic pulmonary disease	12 630	(5.0)	5954	(3.1)	4618	(9.1)	1534	(15.9)	524	(24.9)
Rheumatologic disease	2522	(1.0)	1286	(0.7)	897	(1.8)	261	(2.7)	78	(3.7)
Peptic ulcer disease	7644	(3.0)	2617	(1.4)	3426	(6.7)	1232	(12.8)	369	(17.6)
Mild liver disease	9881	(3.9)	6488	(3.4)	2541	(5.0)	630	(6.5)	222	(10.6)
Diabetes	21 576	(8.5)	13 191	(6.9)	6340	(12.4)	1547	(16.1)	498	(23.7)
Diabetes with chronic complication	6763	(2.7)	3394	(1.8)	2363	(4.6)	726	(7.5)	280	(13.3)
Hemiplegia or paraplegia	586	(0.2)	159	(0.1)	250	(0.5)	117	(1.2)	60	(2.9)
Renal disease	509	(0.2)	124	(0.1)	231	(0.5)	103	(1.1)	51	(2.4)
Malignancy, including leukaemia and lymphoma	8350	(3.3)	5393	(2.8)	2196	(4.3)	605	(6.3)	156	(7.4)
Moderate or severe liver disease	171	(0.1)	82	(0.0)	61	(0.1)	20	(0.2)	8	(0.4)
Metastatic solid tumour	621	(0.2)	303	(0.2)	226	(0.4)	78	(0.8)	14	(0.7)
AIDS	88	(0.0)	65	(0.0)	17	(0.0)	Combined into 6			
Charlson Comorbidity Index										
Mean (SD)	0.4	(0.9)	0.3	(0.7)	0.7	(1.1)	1.2	(1.4)	1.8	(1.6)
0	185 489	(72.9)	153 718	(80.2)	27 861	(54.6)	3474	(36.0)	436	(20.8)
1	45 928	(18.1)	26 467	(13.8)	15 419	(30.2)	3434	(35.6)	608	(28.9)
2	15 322	(6.0)	8393	(4.4)	4920	(9.6)	1528	(15.9)	481	(22.9)
3	5062	(2.0)	2160	(1.1)	1883	(3.7)	708	(7.3)	311	(14.8)
4	1325	(0.5)	376	(0.2)	520	(1.0)	287	(3.0)	142	(6.8)
5+	1205	(0.5)	466	(0.2)	410	(0.8)	206	(2.1)	123	(5.9)
Multimorbidity Frailty Index										
Mean (SD)	0.026	(0.033)	0.010	(0.013)	0.061	(0.012)	0.113	(0.012)	0.179	(0.032)
Aged 65+ years										
Number	138 135		51 179		51 157		24 055		11 744	
Age, years										
Mean (SD)	74.3	(7.0)	72.7	(6.8)	74.6	(7.0)	76.0	(6.9)	77.1	(6.6)
65–74	75 873	(54.9)	33 981	(66.4)	27 172	(53.1)	10 494	(43.6)	4226	(36.0)
75–84	49 478	(35.8)	13 763	(26.9)	19 111	(37.4)	10 673	(44.4)	5931	(50.5)
85+	12 784	(9.3)	3435	(6.7)	4874	(9.5)	2888	(12.0)	1587	(13.5)
Sex										
Male	58 672	(42.5)	23 046	(45.0)	20 938	(40.9)	9588	(39.9)	5100	(43.4)
Female	79 463	(57.5)	28 133	(55.0)	30 219	(59.1)	14 467	(60.1)	6644	(56.6)
Comorbidity										
Myocardial infarction	1420	(1.0)	124	(0.2)	526	(1.0)	436	(1.8)	334	(2.8)
Congestive heart failure	4360	(3.2)	175	(0.3)	1313	(2.6)	1402	(5.8)	1470	(12.5)
Peripheral vascular	7466	(5.4)	1794	(3.5)	2944	(5.8)	1669	(6.9)	1059	(9.0)
Cerebrovascular disease	12 646	(9.2)	1104	(2.2)	4862	(9.5)	3844	(16.0)	2836	(24.1)
Dementia	9024	(6.5)	2154	(4.2)	3351	(6.6)	2099	(8.7)	1420	(12.1)
Chronic pulmonary disease	15 285	(11.1)	2381	(4.7)	5211	(10.2)	4264	(17.7)	3429	(29.2)
Rheumatologic disease	1859	(1.3)	358	(0.7)	701	(1.4)	479	(2.0)	321	(2.7)
Peptic ulcer disease	7281	(5.3)	623	(1.2)	2452	(4.8)	2301	(9.6)	1905	(16.2)
Mild liver disease	4260	(3.1)	1338	(2.6)	1586	(3.1)	827	(3.4)	509	(4.3)
Diabetes	23 444	(17.0)	6683	(13.1)	9107	(17.8)	4861	(20.2)	2793	(23.8)
Diabetes with chronic complication	9417	(6.8)	2010	(3.9)	3634	(7.1)	2325	(9.7)	1448	(12.3)
Hemiplegia or paraplegia	948	(0.7)	73	(0.1)	328	(0.6)	294	(1.2)	253	(2.2)
Renal disease	329	(0.2)	38	(0.1)	105	(0.2)	96	(0.4)	90	(0.8)
Malignancy, including leukaemia and lymphoma	7598	(5.5)	2168	(4.2)	2802	(5.5)	1594	(6.6)	1034	(8.8)
Moderate or severe liver disease	87	(0.1)	24	(0.0)	32	(0.1)	15	(0.1)	16	(0.1)
Metastatic solid tumour	620	(0.4)	166	(0.3)	224	(0.4)	139	(0.6)	91	(0.8)
AIDS	18	(0.0)	8	(0.0)	4	(0.0)	Combined into 6			
Charlson Comorbidity Index										
Mean (SD)	0.9	(1.2)	0.5	(0.9)	0.9	(1.1)	1.3	(1.3)	1.9	(1.6)
0	65 214	(47.2)	33 660	(65.8)	22 338	(43.7)	7209	(30.0)	2007	(17.1)
1	40 908	(29.6)	11 644	(22.8)	17 365	(33.9)	8375	(34.8)	3524	(30.0)
2	18 918	(13.7)	4096	(8.0)	7235	(14.1)	4705	(19.6)	2882	(24.5)
3	8165	(5.9)	1287	(2.5)	2850	(5.6)	2324	(9.7)	1704	(14.5)
4	2941	(2.1)	256	(0.5)	834	(1.6)	922	(3.8)	929	(7.9)
5+	1989	(1.4)	236	(0.5)	535	(1.0)	520	(2.2)	698	(5.9)
Multimorbidity Frailty Index										
Mean (SD)	0.066	(0.053)	0.017	(0.013)	0.064	(0.013)	0.115	(0.013)	0.187	(0.038)

Sex distribution patterns revealed important cross‐national differences in frailty development. In Taiwan, men represented a significantly higher proportion of severely frail individuals in both age groups (61.4% of severely frail middle‐aged and 53.9% of severely frail older adults), while South Korea showed less variation in sex distribution across frailty categories.

The comorbidity profiles demonstrated strong associations with frailty severity. In Taiwan, the mean CCI increased from 0.4 ± 1.0 in fit middle‐aged adults to 3.3 ± 2.4 in severely frail individuals (*p* < 0.001), with those having a CCI of 5+ rising dramatically from 0.9% to 27.4%. The mFI showed corresponding increases (from 0.009 ± 0.012 to 0.175 ± 0.027), validating this tool across both Asian populations. Specific comorbidities showed marked prevalence gradients, with congestive heart failure increasing from 0.2% in fit to 24.7% in severely frail middle‐aged Taiwanese, and peptic ulcer disease reaching 44.0% in the severely frail group—the highest prevalence of any comorbidity.

### 3.2 Healthcare Utilisation Patterns Across Frailty Status

**FIGURE 1:**
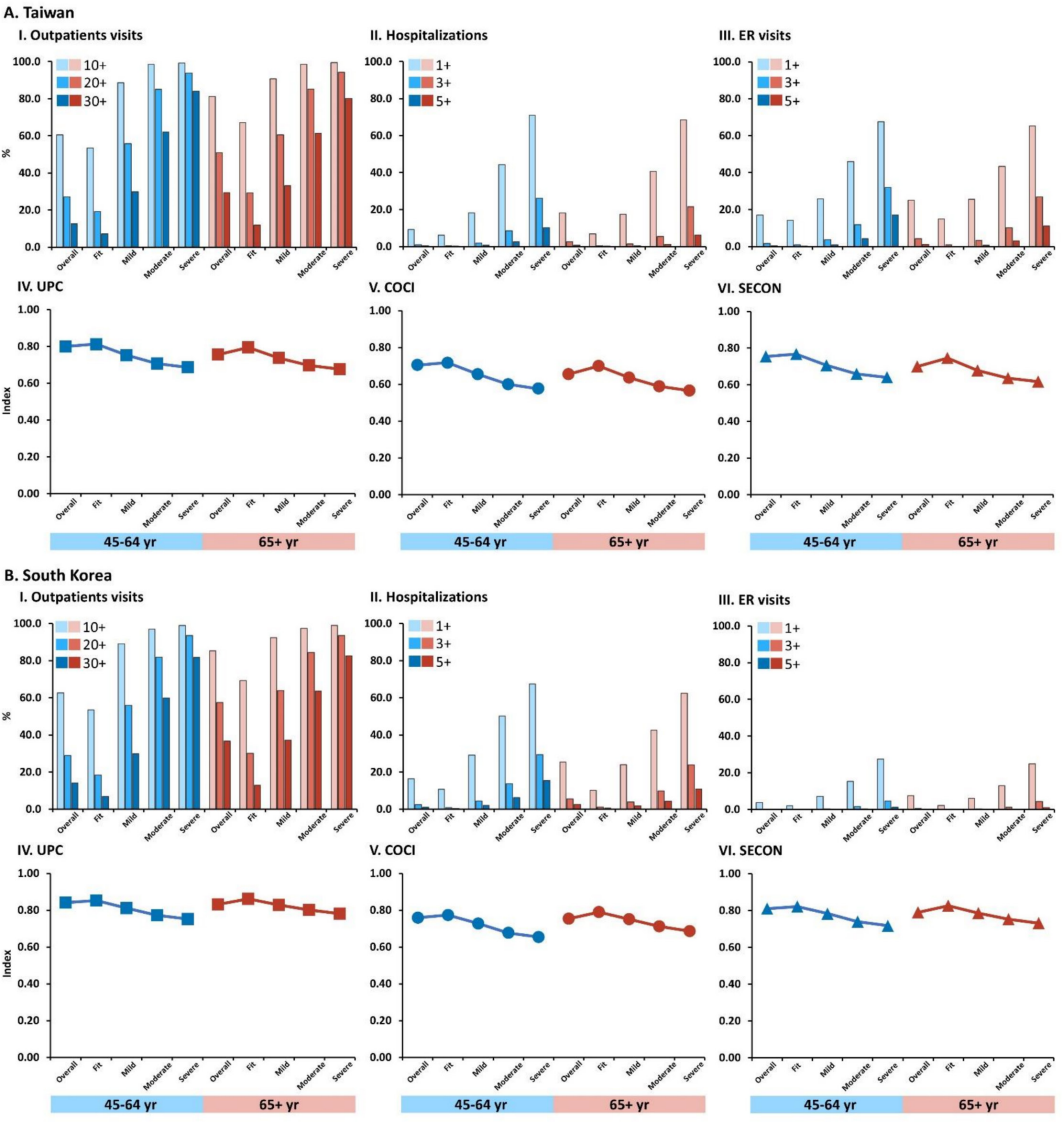
Baseline healthcare utilisation and healthcare fragmentation of study participants stratified by frailty status. ER, emergency room; COCI, Continuity of Care Index; SECON, Sequential Continuity Index; UPC, Usual Provider of Care Index.

Healthcare utilisation increased progressively with frailty severity across all metrics in both countries (*p* < 0.001), revealing the substantial clinical and economic burden of frailty (Figure 1, Tables S1–S4).

In Taiwan, middle‐aged severely frail individuals had 4.3 times more annual outpatient visits than their fit counterparts (56.5 ± 31.0 vs. 13.2 ± 10.4, *p* < 0.001), with 84.1% requiring 30 + visits annually compared to just 7.2% of fit adults. This gradient was even steeper in South Korea, where middle‐aged severely frail adults had five times more outpatient visits than fit individuals (67.1 ± 55.1 vs. 13.3 ± 11.7 visits, *p* < 0.001), with 81.7% requiring 30+ visits compared to only 6.9% of fit adults. Among older adults, the pattern was similar in both countries, with severely frail individuals having approximately three times more outpatient visits than their fit counterparts.

The clinical complexity of caring for frail individuals was evident in the number of involved providers and transitions between them. The mean number of involved providers increased significantly from fit to severely frail groups in both countries and age categories (*p* < 0.001). In Taiwan, middle‐aged severely frail individuals consulted 2.7 ± 0.8 different providers annually compared to 1.9 ± 0.8 for fit individuals. More remarkably, provider transitions increased nearly sevenfold from fit to severely frail middle‐aged Taiwanese (3.0 ± 4.2 to 19.8 ± 14.4, *p* < 0.001). Among older adults, transitions rose from 4.2 ± 5.2 in the fit group to 18.9 ± 13.0 in the severely frail group (*p* < 0.001). South Korea showed similar patterns, with provider transitions increasing from 2.1 ± 3.3 to 16.8 ± 16.3 in middle‐aged adults across the frailty spectrum (*p* < 0.001). Our findings demonstrate that care fragmentation increases proportionally with frailty, creating a paradoxical situation wherein those most requiring coordinated care experience the greatest discontinuity—a phenomenon with significant implications for both care quality outcomes and healthcare expenditure.

Hospital and ED utilisation showed the most striking differences. In Taiwan, 70.9% of severely frail middle‐aged adults required hospitalisation annually versus 6.1% of fit individuals, with those needing 3+ admissions rising from 0.5% to 26.1% (*p* < 0.001). LOS demonstrated a statistically significant increase of 158.4%, from 10.1 ± 25.6 days to 26.1 ± 37.0 days (*p* < 0.001), suggesting substantial implications for resource utilisation and clinical pathways. In South Korea, the hospitalisation pattern was similar, with 67.5% of severely frail middle‐aged adults requiring hospitalisation compared to 10.8% of fit individuals (*p* < 0.001), and those with 3 + admissions increasing from 0.9% to 29.4% (p < 0.001). Notably, South Korean patients had substantially longer LOS across all frailty categories, with severely frail middle‐aged adults averaging 37.0 ± 67.9 days compared to 26.1 ± 37.0 days in Taiwan (*p* < 0.001).

ED utilisation showed marked cross‐national differences, with Taiwan reporting substantially higher ED use than South Korea across all frailty categories. Among severely frail middle‐aged adults, 67.5% in Taiwan had ED visits versus only 27.5% in South Korea (*p* < 0.001), suggesting different approaches to urgent care delivery and potential opportunities for system‐level interventions to reduce high‐intensity service use.

### 3.3 Care Fragmentation by Frailty Status

Care fragmentation increased significantly with frailty severity in both countries (*p* < 0.001 for all trends), demonstrating that those with the most complex needs paradoxically experienced the most dispersed care (Figure 1, Tables S1–S4).

In Taiwan, all three continuity indices showed progressive deterioration with increasing frailty. Among middle‐aged adults, the UPC decreased from 0.8128 ± 0.1842 in the fit group to 0.6868 ± 0.1698 in the severely frail group, indicating a 15.5% reduction in care concentration with a single provider. Similarly, the COCI decreased from 0.7171 ± 0.2544 to 0.5759 ± 0.1839 (19.7% reduction), and the SECON from 0.7673 ± 0.2344 to 0.6403 ± 0.1810 (16.6% reduction). In South Korea, similar but less pronounced trends were observed, with UPC decreasing from 0.8530 ± 0.1719 to 0.7524 ± 0.1683 (11.8% reduction), COCI from 0.7736 ± 0.2441 to 0.6544 ± 0.1951 (15.4% reduction) and SECON from 0.8222 ± 0.2158 to 0.7179 ± 0.1771 (12.7% reduction) among middle‐aged adults.

The observed pattern of care fragmentation persisted across older populations in both healthcare systems; however, noteworthy cross‐national variations emerged, suggesting the influence of distinct systemic factors and policy frameworks on care coordination pathways. Older Taiwanese adults showed lower continuity indices overall and steeper declines with frailty compared to their South Korean counterparts. The UPC for severely frail older Taiwanese was 0.6759 ± 0.1666 compared to 0.7813 ± 0.1595 for severely frail older South Koreans (*p* < 0.001), and similar differences were observed in COCI (0.5654 ± 0.1818 vs. 0.6860 ± 0.1891, *p* < 0.001) and SECON (0.6174 ± 0.1846 vs. 0.7314 ± 0.1787, *p* < 0.001). These cross‐national differences, consistent across all continuity metrics and frailty categories, suggest structural healthcare system differences that may affect care coordination independently of patient characteristics, with potential implications for the development of targeted interventions to improve care continuity for frail individuals.

### 3.4 Association Between Healthcare Utilisation and Mortality

During the 3‐year follow‐up period, mortality increased markedly with age and frailty severity in both countries. Among adults younger than 65 years, 3‐year mortality ranged from 1.7% in Taiwan and 1.0% in South Korea overall, rising from 1.2% and 0.7% in fit individuals to 16.6% and 6.4% in those with severe frailty, respectively. Among adults aged 65 years or older, overall 3‐year mortality was 10.7% in Taiwan and 8.1% in South Korea, increasing from 5.9% and 5.4% in fit individuals to 31.8% and 16.3% in those with severe frailty.

**FIGURE 2:**
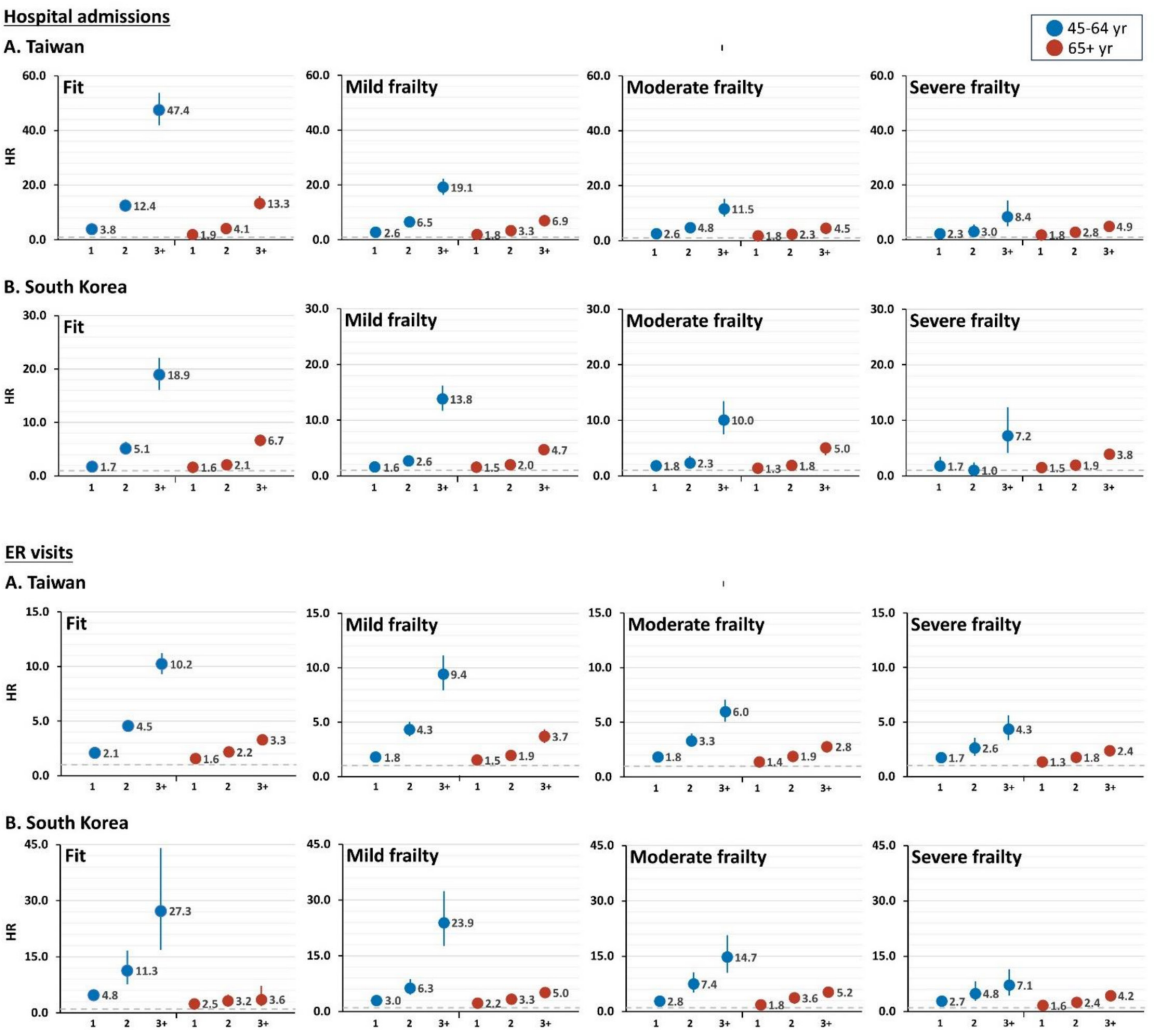
All‐cause mortality by healthcare utilisation stratified by frailty status. Reference for healthcare utilisation indicator = 0 Adjusted for age and sex HR, hazard ratio.

Our analysis revealed a significant but nuanced relationship between healthcare utilisation and mortality. In both countries, increased healthcare utilisation was associated with higher mortality risk; however, the strength of this association weakened substantially with increasing frailty severity. In Taiwan, middle‐aged fit individuals with ≥ 3 hospitalisations had a 47.42‐fold increased mortality risk (95% CI 41.87–53.71) compared to those without hospitalisations, whereas severely frail individuals with similar utilisation had only an 8.44‐fold increased risk (95% CI 4.96–14.35) (Figure 2).

### 3.5 Association Between Care Fragmentation and Mortality

**FIGURE 3:**
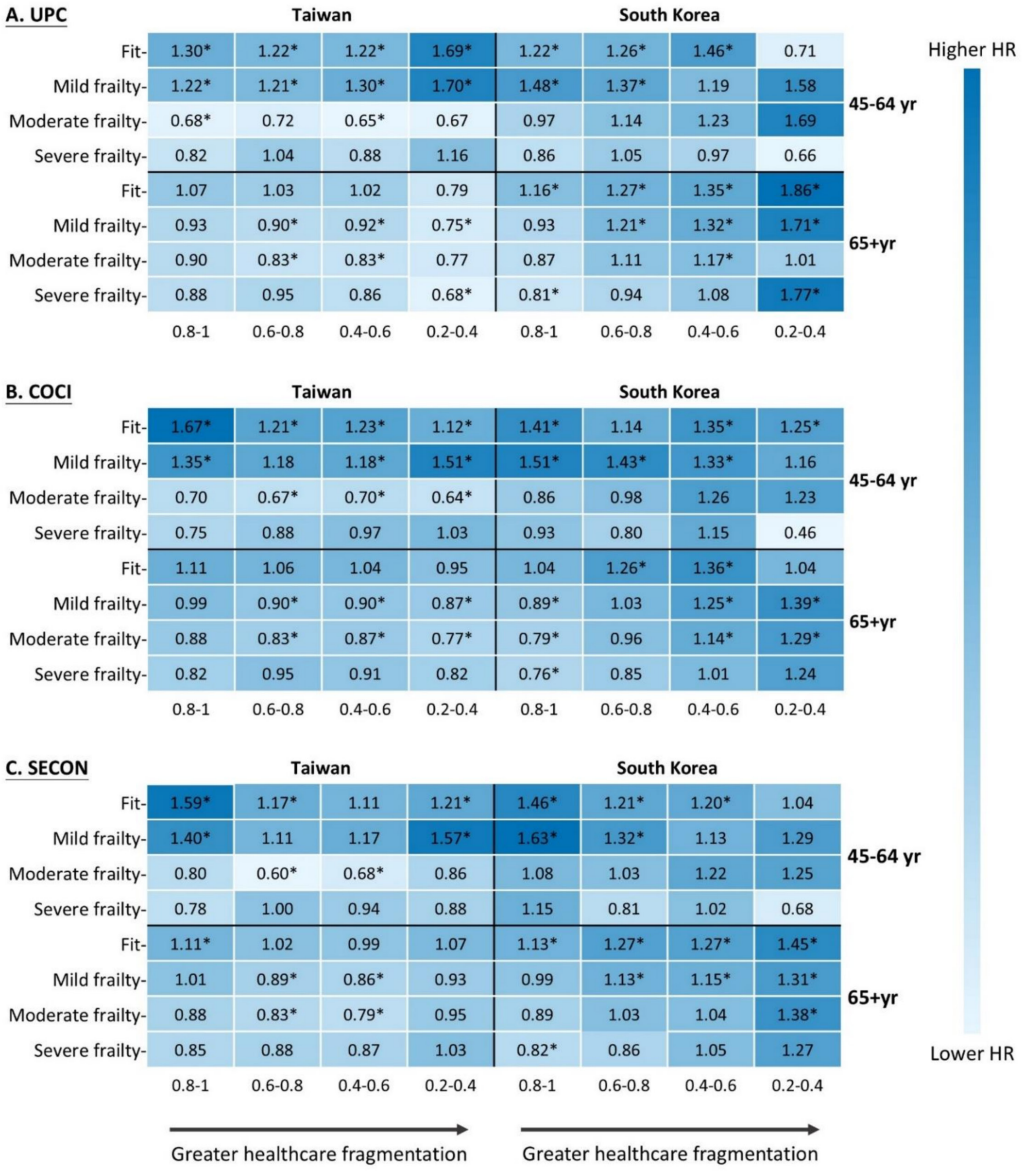
All‐cause mortality by care fragmentation indices stratified by frailty status. Reference for care fragmentation indicator = 1. Adjusted for age and sex. HR, hazard ratio; COCI, Continuity of Care Index; SECON, Sequential Continuity Index; UPC, Usual Provider of Care Index. *With statistically significance. Due to the limited number of individuals in the 0–0.2 index group, this category was not displayed in the figure.

The most significant finding of this study was the complex, bidirectional relationship between care fragmentation and mortality across the frailty spectrum. In both countries, higher care fragmentation was associated with significantly increased mortality risk in fit individuals but showed no association or even inverse effects in those with severe frailty. In Taiwan, fit middle‐aged adults with a UPC index of 0.2–0.4 had a 69% higher mortality risk (aHR 1.69, 95% CI 1.14–2.50, *p* < 0.01) compared to those with a UPC of 1, whereas moderately frail individuals with the same UPC level showed a nonsignificant 33% lower risk (aHR 0.67, 95% CI 0.31–1.42) (Figure 3).

## 4 Discussion

This cross‐national investigation demonstrates that frailty fundamentally transforms the relationship between healthcare utilisation, care fragmentation and clinical outcomes. Comparing Taiwan and South Korea, we observed a universal paradoxical phenomenon: fragmented care strongly predicted mortality in fit individuals but showed inverse associations in severely frail populations. This finding, consistent across both countries, challenges the prevailing assumption that care continuity universally benefits all patients. The fundamental relationships between frailty, care fragmentation and mortality followed similar patterns in both countries. Notably, despite lower frailty prevalence in middle‐aged adults compared to older adults, the impact of severe frailty on healthcare utilisation was more pronounced in the middle‐aged cohort. The fragmentation‐mortality paradox persisted across both age groups in both countries, suggesting that frailty, rather than chronological age, should guide healthcare delivery models. These findings demonstrate the necessity for a significant reorientation in healthcare quality conceptualisation for vulnerable adults with complex care needs, moving toward frailty‐specific coordination strategies that recognise the complex, non‐linear relationships between frailty status, care models and outcomes in ageing populations.

Frailty represents a critical phenotype that fundamentally transforms healthcare needs, health‐social care utilisation and outcome determinants [33, 34]. Unlike traditional disease‐based classification systems, frailty assessment provides a multidimensional framework that captures physiological vulnerability of older individuals [35]. The stark utilisation differentials across frailty categories—with severely frail individuals requiring exponentially more healthcare utilisation and provider interactions—highlights frailty's utility as a healthcare resource planning tool [36]. Quality improvement initiatives should therefore incorporate frailty assessment in the evaluation frameworks [37], as traditional continuity metrics may not sufficiently capture care quality for those with complex multimorbidity requiring specialised, multidisciplinary approaches. To contextualise our findings within the existing evidence base, frailty emerges as the critical framework through which health service efficiency, quality and resource allocation must be assessed—particularly germane to rapidly ageing societies where population‐level frailty prevalence continues to rise. Most European countries have established and implemented specialised geriatric services for decades [38], whereas Taiwan, South Korea and most Asian countries have not yet developed comparable infrastructure [39]; thus, the inverse relationship between care fragmentation and mortality in severely frail older adults may indeed reflect the insufficiency of nationwide frailty service frameworks and the absence of accessible specialised geriatric care models.

Our analysis employed multiple continuity indices to capture the multifaceted nature of care coordination, each revealing distinct dimensions of care delivery. The UPC demonstrated the steepest deterioration with increasing frailty, reflecting patients' tendency to distribute care across multiple specialists as complexity increased [40]. The COCI provided nuanced insights into care distribution patterns [41], whereas the SECON highlighted the temporal dynamics of provider transitions [42]. Importantly, despite measuring different aspects of continuity, all indices converged in revealing the paradoxical relationship between care fragmentation and mortality across frailty categories. This convergent validity across multiple metrics strengthens our findings that the fragmentation‐mortality association fundamentally transforms with frailty status. In Taiwan, fit individuals with low continuity showed 69% higher mortality risk, whereas frail individuals with identical scores demonstrated a nonsignificant 33% lower risk—a pattern replicated in South Korea. This study transcends conventional frailty research on healthcare utilisation by elucidating structural healthcare differences between countries and their impacts on fragmentation‐mortality dynamics [43].

Although a previous Danish study demonstrated an association between healthcare provider transitions and elevated mortality [28], our findings elucidate the more nuanced mediating role of frailty—particularly severe frailty—in fundamentally transforming healthcare system efficiency, quality and outcome relationships. The single‐payer systems of Taiwan and South Korea, with less established gatekeeping mechanisms, generate notably high provider transition rates among severely frail individuals; however, the absence of universally implemented frailty service frameworks confounds the putative beneficial effects of care continuity within these healthcare systems. For severely frail patients with complex care needs, distributed care across specialists may represent expertise‐seeking behaviours in systems lacking accessible specialised geriatric services. The steeper decline in continuity indices with increasing frailty in Taiwan compared to South Korea highlights how delivery system differences influence care patterns despite similar financing structures. Healthcare systems should develop frailty‐specific coordination mechanisms and accessible specialised geriatric services with information continuity.

Health‐seeking behaviour reflects a multidimensional interplay between individual characteristics (socioeconomic status, health literacy and symptom perception), social–cultural influences (family dynamics and cultural beliefs) and healthcare system structures (accessibility, gatekeeping mechanisms and financial incentives) [44]. The paradoxical relationship identified in this study parallels research on polypharmacy—where despite generally being associated with adverse outcomes, the strength of this association was significantly attenuated in older people with severe frailty [45]. Collectively, these findings underscore the urgent necessity for a systematic frailty service framework and accessible specialised geriatric services that integrate multidisciplinary expertise, rather than merely emphasising enhanced care continuity as the primary metric of quality. Improving outcomes for frail populations necessitates more comprehensive information sharing, robust clinical decision support systems and specialised frailty expertise embedded throughout care pathways. Healthcare systems with well‐founded primary care gatekeeping mechanisms and established geriatric service infrastructures may exhibit different fragmentation‐mortality associations, as structured care coordination could potentially attenuate the paradoxical relationship observed in severely frail populations by enabling appropriate multidisciplinary consultations while preserving informational and management continuity across care transitions.

This study has several limitations that warrant consideration. First, our reliance on retrospective claims data may introduce potential biases related to incomplete records or misclassification of frailty status. In addition, as is common in claims‐based databases, direct measures of disability, functional dependence and loss of autonomy were unavailable. Although the multimorbidity frailty index captures accumulated health deficits and has been validated in relation to mortality, it does not directly reflect functional impairment or caregiving needs. The absence of such information may have influenced observed patterns of healthcare utilisation, care fragmentation and mortality. Second, despite using standardised measures, cross‐national comparisons may still be confounded by unmeasured differences in healthcare systems, diagnostic coding practices and cultural factors influencing care‐seeking behaviours. Third, the absence of a nationwide frailty service framework and widely implemented specialised geriatric services in both countries precluded validation of these services' potential to reverse the fragmentation‐mortality paradox observed in severely frail older adults. Nevertheless, our study possesses considerable strengths that enhance the reliability and applicability of the findings. First, the large, nationally representative sample size significantly improves the precision and generalisability of our results. Second, the comprehensive analysis of frailty across multiple dimensions—demographics, healthcare utilisation patterns, care continuity metrics and mortality outcomes—provides a multifaceted understanding of frailty's impact. Furthermore, employing consistent measurements for frailty, continuity of care and definitions of healthcare utilisation across two distinct healthcare systems strengthens the cross‐cultural applicability of our findings and suggests the potential for broader international implementation.

## 5 Conclusions

Our cross‐national study identifies the fragmentation‐mortality paradox in frail middle‐aged and older adults, which reflects the urgent need for a systematic frailty service framework and accessible specialised geriatric services. Policy reform requires standardised frailty assessment frameworks in routine care systems and substantial investment in a specialised geriatric workforce, enabling healthcare systems toward interdisciplinary models with comprehensive integration.

## Funding

We thank the National Health Insurance Administration (NHIA) and Health and Welfare Data Science Center (HWDC) for making the databases used in this study available; however, the content of this article does not represent any official position of the NHIA or HWDC. The authors had full access to all of the data in this study and take responsibility for the integrity of the data and the accuracy of the data analysis. This study was supported by the National Science and Technology Council, Taiwan (NSTC 114‐2321‐B‐A49‐012‐). This work was also supported by the Interdisciplinary Research Center for Healthy Longevity of National Yang Ming Chiao Tung University from The Featured Areas Research Center Program within the framework of the Higher Education Sprout Project by the Ministry of Education (MOE) in Taiwan. The funding agencies played no role in the study design, data collection, analysis, interpretation or report writing.

## Conflicts of Interest

The authors declare no conflicts of interest.

## Supporting information


**Figure S1:** Flow chart of Taiwanese cohort.
**Figure S2:** Flow chart of South Korean cohort.
**Table S1:** Baseline healthcare utilisation and healthcare fragmentation of study participants aged 45 to 64 years in Taiwan, stratified by frailty status.
**Table S2:** Baseline healthcare utilisation and healthcare fragmentation of study participants aged 65+ years in Taiwan, stratified by frailty status.
**Table S3:** Baseline healthcare utilisation and healthcare fragmentation of study participants aged 45 to 64 years in South Korea, stratified by frailty status.
**Table S4:** Baseline healthcare utilisation and healthcare fragmentation of study participants aged 65+ years in South Korea, stratified by frailty status.
**Table S5:** Hazard ratio of all‐cause mortality among study participants aged 45–64 years in Taiwan by fitting Cox regression model, stratified by frailty status.
**Table S6:** Hazard ratio of all‐cause mortality among study participants aged 65+ years in Taiwan by fitting Cox regression model, stratified by frailty status.
**Table S7:** Hazard ratio of all‐cause mortality among study participants aged 45–64 years in South Korea by fitting Cox regression model, stratified by frailty status.
**Table S8:** Hazard ratio of all‐cause mortality among study participants aged 65+ years in South Korea by fitting Cox regression model, stratified by frailty status.

## Data Availability

All potentially identifiable data were encrypted to protect anonymity. Access to data is restricted to investigators whose proposal is approved and who have signed a data access agreement.
