# Supplementary material for: Frailty Transforms Care Continuity‐Mortality Relationships Across Age Groups: Evidence From Taiwan and South Korea
Source: J Cachexia Sarcopenia Muscle. 2026 May 14;17(3):e70303. doi: 10.1002/jcsm.70303 (PMC13176647; doi:10.1002/jcsm.70303)
Supplement: Supplementary file 1 — Figure S1: Flow chart of Taiwanese cohort. Figure S2: Flow chart of South Korean cohort. Table S1: Baseline healthcare utilisation and healthcare fragmentation of study participants aged 45 to 64 years in Taiwan, stratified by frailty status. Table S2: Baseline healthcare utilisation and healthcare fragmentation of study participants aged 65+ years in Taiwan, stratified by frailty status. Table S3: Baseline healthcare utilisation and healthcare fragmentation of study participants aged 45 to 64 years in South Korea, stratified by frailty status. Table S4: Baseline healthcare utilisation and healthcare fragmentation of study participants aged 65+ years in South Korea, stratified by frailty status. Table S5: Hazard ratio of all‐cause mortality among study participants aged 45–64 years in Taiwan by fitting Cox regression model, stratified by frailty status. Table S6: Hazard ratio of all‐cause mortality among study participants aged 65+ years in Taiwan by fitting Cox regression model, stratified by frailty status. Table S7: Hazard ratio of all‐cause mortality among study participants aged 45–64 years in South Korea by fitting Cox regression model, stratified by frailty status. Table S8: Hazard ratio of all‐cause mortality among study participants aged 65+ years in South Korea by fitting Cox regression model, stratified by frailty status. [file JCSM-17-e70303-s001.docx]

**Supplementary Data**

**Frailty transforms care continuity-mortality relationships across age groups: Evidence from Taiwan and South Korea**

**Supplementary Figures**

eFigure 1. Flow chart of Taiwanese cohort

eFigure 2. Flow chart of South Korean cohort

**Supplementary Tables**

eTable 1. Baseline healthcare utilization and healthcare fragmentation of study participants aged 45 to 64 years in Taiwan, stratified by frailty status

eTable 2. Baseline healthcare utilization and healthcare fragmentation of study participants aged 65+ years in Taiwan, stratified by frailty status

eTable 3. Baseline healthcare utilization and healthcare fragmentation of study participants aged 45 to 64 years in South Korea, stratified by frailty status

eTable 4. Baseline healthcare utilization and healthcare fragmentation of study participants aged 65+ years in South Korea, stratified by frailty status

eTable 5. Hazard ratio of all-cause mortality among study participants aged 45-64 years in Taiwan by fitting Cox regression model, stratified by frailty status

eTable 6. Hazard ratio of all-cause mortality among study participants aged 65+ years in Taiwan by fitting Cox regression model, stratified by frailty status

eTable 7. Hazard ratio of all-cause mortality among study participants aged 45-64 years in South Korea by fitting Cox regression model, stratified by frailty status

eTable 8. Hazard ratio of all-cause mortality among study participants aged 65+ years in South Korea by fitting Cox regression model, stratified by frailty status

**eFigure 1. Flow chart of the Taiwanese cohort**

Study population, N = 370,997

|  | Age 45-64 | Age 65+ |
| --- | --- | --- |
| Fit | 187,945 (80.8%) | 63,877 (46.2%) |
| Mild frailty | 38,321 (16.5%) | 49,513 (35.8%) |
| Moderate frailty | 5,341 (2.3%) | 17,602 (12.7%) |
| Severe frailty | 1,040 (0.4%) | 7,358 (5.3%) |

A subset consisting of 1,000,000 beneficiaries

Exclude:

- Subjects aged less than 45 years (n=542,564)

- Subjects died before December 31, 2017 (n=6,504)

- Subjects without at least three outpatient visit in 2017 (n=79,935)

**eFigure 2. Flow chart of the South Korean cohort**

Study population, N = 392,466

|  | Age 45-64 | Age 65+ |
| --- | --- | --- |
| Fit | 191,580 (75.3%) | 51,179 (37.1%) |
| Mild frailty | 51,013 (20.1%) | 51,157 (37.0%) |
| Moderate frailty | 9,637 (3.8%) | 24,055 (17.4%) |
| Severe frailty | 2,101 (0.8%) | 11,744 (8.5%) |

A subset consisting of 1,000,000 beneficiaries

Exclude:

- Subjects aged less than 45 years (n=530,218)

- Subjects died before December 31, 2017 (n=7)

- Subjects without at least three outpatient visit in 2017 (n=76,309)

**eTable 1. Baseline healthcare utilization and healthcare fragmentation of study participants aged 45 to 64 years in Taiwan, stratified by frailty status**

|  | | | *Total (N=*232,647*)* | | *Fit (N=*187,945*)* | | *Mild frailty (N=*38,321*)* | | *Moderate frailty (N=*5,341*)* | | *Severe frailty (N=*1,040*)* | |
| --- | --- | --- | --- | --- | --- | --- | --- | --- | --- | --- | --- | --- |
|  | | | *n* | *(%)* | *n* | *(%)* | *n* | *(%)* | *n* | *(%)* | *n* | *(%)* |
| **Outpatient visit** | | | | | | | | | | | | |
|  | Number of visits | Mean (SD) | 15.9 | (13.6) | 13.2 | (10.4) | 25.1 | (16.1) | 39.6 | (22.6) | 56.5 | (31.0) |
|  | 10+ visits |  | 140866 | (60.5) | 100631 | (53.5) | 33946 | (88.6) | 5258 | (98.4) | 1031 | (99.1) |
|  | 20+ visits |  | 63145 | (27.1) | 36233 | (19.3) | 21388 | (55.8) | s4547 | (85.1) | 977 | (93.9) |
|  | 30+ visits |  | 29182 | (12.5) | 13561 | (7.2) | 11436 | (29.8) | 3310 | (62.0) | 875 | (84.1) |
|  | Involved providers | Mean (SD) | 1.9 | (0.8) | 1.9 | (0.8) | 2.2 | (0.8) | 2.5 | (0.8) | 2.7 | (0.8) |
|  | Provider transitions | Mean (SD) | 4.0 | (5.7) | 3.0 | (4.2) | 7.3 | (7.5) | 13.1 | (10.9) | 19.8 | (14.4) |
| **Hospitalization** | | | | | | | | | | | | |
|  | Number of hospitalizations | Mean (SD) | 0.1 | (0.7) | 0.1 | (0.5) | 0.3 | (0.9) | 0.8 | (1.5) | 1.9 | (2.3) |
|  | 1+ admissions |  | 21595 | (9.3) | 11472 | (6.1) | 7015 | (18.3) | 2371 | (44.4) | 737 | (70.9) |
|  | 3+ admissions |  | 2388 | (1.0) | 887 | (0.5) | 777 | (2.0) | 453 | (8.5) | 271 | (26.1) |
|  | 5+ admissions |  | 986 | (0.4) | 445 | (0.2) | 299 | (0.8) | 136 | (2.5) | 106 | (10.2) |
|  | Hospital length of stay | Mean (SD) | 12.5 | (28.0) | 10.1 | (25.6) | 13.2 | (28.8) | 17.4 | (31.4) | 26.1 | (37.0) |
| **ER visit** | | | | | | | | | | | | |
|  | Number of ER visits | Mean (SD) | 0.3 | (1.0) | 0.2 | (0.6) | 0.4 | (1.2) | 1.1 | (3.3) | 2.9 | (5.8) |
|  | 1+ visits |  | 39572 | (17.0) | 26554 | (14.1) | 9861 | (25.7) | 2455 | (46.0) | 702 | (67.5) |
|  | 3+ visits |  | 4057 | (1.7) | 1652 | (0.9) | 1435 | (3.7) | 637 | (11.9) | 333 | (32.0) |
|  | 5+ visits |  | 1097 | (0.5) | 305 | (0.2) | 384 | (1.0) | 229 | (4.3) | 179 | (17.2) |
| **Fragmentation indices** | | | | | | | | | | | | |
|  | Usual Provider of Care Index (UPC) | Mean (SD) | 0.7998 | (0.1859) | 0.8128 | (0.1842) | 0.7522 | (0.1836) | 0.7070 | (0.1732) | 0.6868 | (0.1698) |
|  | Continuity Of Care Index (COCI) | Mean (SD) | 0.7034 | (0.2499) | 0.7171 | (0.2544) | 0.6537 | (0.2235) | 0.5994 | (0.1961) | 0.5759 | (0.1839) |
|  | Sequential Continuity Index (SECON) | Mean (SD) | 0.7542 | (0.2318) | 0.7673 | (0.2344) | 0.7063 | (0.2147) | 0.6594 | (0.1917) | 0.6403 | (0.1810) |

**eTable 2. Baseline healthcare utilization and healthcare fragmentation of study participants aged 65+ years in Taiwan, stratified by frailty status**

|  | | | *Total (N=*138,350*)* | | *Fit (N=*63,877*)* | | *Mild frailty (N=*49,513*)* | | *Moderate frailty (N=*17,602*)* | | *Severe frailty (N=*7,358*)* | |
| --- | --- | --- | --- | --- | --- | --- | --- | --- | --- | --- | --- | --- |
|  | | | *n* | *(%)* | *n* | *(%)* | *n* | *(%)* | *n* | *(%)* | *n* | *(%)* |
| **Outpatient visit** | | | | | | | | | | | | |
|  | Number of visits | Mean (SD) | 24.5 | (18.3) | 16.3 | (12.0) | 26.4 | (16.1) | 38.1 | (19.6) | 50.5 | (24.8) |
|  | 10+ visits |  | 112373 | (81.2) | 42782 | (67.0) | 44923 | (90.7) | 17348 | (98.6) | 7320 | (99.5) |
|  | 20+ visits |  | 70564 | (51.0) | 18674 | (29.2) | 29946 | (60.5) | 15002 | (85.2) | 6942 | (94.3) |
|  | 30+ visits |  | 40684 | (29.4) | 7602 | (11.9) | 16391 | (33.1) | 10790 | (61.3) | 5901 | (80.2) |
|  | Involved providers | Mean (SD) | 2.2 | (0.8) | 2.0 | (0.8) | 2.3 | (0.8) | 2.5 | (0.8) | 2.7 | (0.8) |
|  | Provider transitions | Mean (SD) | 7.6 | (8.6) | 4.2 | (5.2) | 8.4 | (7.8) | 13.5 | (10.2) | 18.9 | (13.0) |
| **Hospitalization** | | | | | | | | | | | | |
|  | Number of hospitalizations | Mean (SD) | 0.3 | (0.9) | 0.1 | (0.5) | 0.3 | (0.8) | 0.7 | (1.1) | 1.5 | (1.7) |
|  | 1+ admissions |  | 25151 | (18.2) | 4315 | (6.8) | 8637 | (17.4) | 7152 | (40.6) | 5047 | (68.6) |
|  | 3+ admissions |  | 3513 | (2.5) | 288 | (0.5) | 687 | (1.4) | 950 | (5.4) | 1588 | (21.6) |
|  | 5+ admissions |  | 998 | (0.7) | 132 | (0.2) | 208 | (0.4) | 204 | (1.2) | 454 | (6.2) |
|  | Hospital length of stay | Mean (SD) | 13.9 | (24.7) | 9.6 | (20.4) | 11.2 | (22.2) | 13.9 | (23.6) | 22.2 | (31.1) |
| **ER visit** | | | | | | | | | | | | |
|  | Number of ER visits | Mean (SD) | 0.5 | (1.3) | 0.2 | (0.6) | 0.4 | (1.2) | 0.9 | (1.6) | 1.9 | (2.9) |
|  | 1+ visits |  | 34559 | (25.0) | 9446 | (14.8) | 12671 | (25.6) | 7638 | (43.4) | 4804 | (65.3) |
|  | 3+ visits |  | 5979 | (4.3) | 579 | (0.9) | 1637 | (3.3) | 1787 | (10.2) | 1976 | (26.9) |
|  | 5+ visits |  | 1825 | (1.3) | 90 | (0.1) | 369 | (0.7) | 541 | (3.1) | 825 | (11.2) |
| **Fragmentation indices** | | | | | | | | | | | | |
|  | Usual Provider of Care Index (UPC) | Mean (SD) | 0.7550 | (0.1857) | 0.7945 | (0.1851) | 0.7368 | (0.1815) | 0.6961 | (0.1721) | 0.6759 | (0.1666) |
|  | Continuity Of Care Index (COCI) | Mean (SD) | 0.6549 | (0.2302) | 0.6988 | (0.2443) | 0.6352 | (0.2170) | 0.5880 | (0.1940) | 0.5654 | (0.1818) |
|  | Sequential Continuity Index (SECON) | Mean (SD) | 0.7000 | (0.2242) | 0.7450 | (0.2305) | 0.6772 | (0.2176) | 0.6357 | (0.1975) | 0.6174 | (0.1846) |

**eTable 3. Baseline healthcare utilization and healthcare fragmentation of study participants aged 45 to 64 years in South Korea, stratified by frailty status**

|  | | | *Total* (N=254,331) | | *Fit (N=*191,580*)* | | *Mild frailty (N=*51,013*)* | | *Moderate frailty (N=*9,637*)* | | *Severe frailty (N=*2,101*)* | |
| --- | --- | --- | --- | --- | --- | --- | --- | --- | --- | --- | --- | --- |
|  | | | *n* | *(%)* | *n* | *(%)* | *n* | *(%)* | *n* | *(%)* | *n* | *(%)* |
| **Outpatient visit** | | | | | | | | | | | | |
|  | Number of visits | Mean (SD) | 17.4 | (18.2) | 13.3 | (11.7) | 26.4 | (21.0) | 42.4 | (32.3) | 67.1 | (55.1) |
|  | 10+ visits |  | 159313 | (62.6) | 102453 | (53.5) | 45434 | (89.1) | 9346 | (97.0) | 2080 | (99.0) |
|  | 20+ visits |  | 73755 | (29.0) | 35394 | (18.5) | 28508 | (55.9) | 7888 | (81.9) | 1965 | (93.5) |
|  | 30+ visits |  | 35946 | (14.1) | 13253 | (6.9) | 15201 | (29.8) | 5775 | (59.9) | 1717 | (81.7) |
|  | Involved providers | Mean (SD) | 1.8 | (0.7) | 1.7 | (0.7) | 2.0 | (0.7) | 2.2 | (0.7) | 2.4 | (0.7) |
|  | Provider transitions | Mean (SD) | 3.1 | (5.0) | 2.1 | (3.3) | 5.2 | (6.0) | 9.6 | (9.2) | 16.8 | (16.3) |
| **Hospitalization** | | | | | | | | | | | | |
|  | Number of hospitalizations | Mean (SD) | 0.3 | (1.3) | 0.2 | (0.8) | 0.6 | (1.8) | 1.3 | (2.6) | 2.5 | (3.8) |
|  | 1+ admissions |  | 41812 | (16.4) | 20724 | (10.8) | 14828 | (29.1) | 4841 | (50.2) | 1419 | (67.5) |
|  | 3+ admissions |  | 6020 | (2.4) | 1807 | (0.9) | 2272 | (4.5) | 1323 | (13.7) | 618 | (29.4) |
|  | 5+ admissions |  | 2703 | (1.1) | 756 | (0.4) | 1020 | (2.0) | 604 | (6.3) | 323 | (15.4) |
|  | Hospital length of stay | Mean (SD) | 16.1 | (43.9) | 11.9 | (35.4) | 17.3 | (46.5) | 24.2 | (54.8) | 37.0 | (67.9) |
| **ER visit** | | | | | | | | | | | | |
|  | Number of ER visits | Mean (SD) | 0.0 | (0.3) | 0.0 | (0.2) | 0.1 | (0.4) | 0.2 | (0.7) | 0.5 | (1.1) |
|  | 1+ visits |  | 9565 | (3.8) | 3862 | (2.0) | 3642 | (7.1) | 1484 | (15.4) | 577 | (27.5) |
|  | 3+ visits |  | 523 | (0.2) | 92 | (0.0) | 171 | (0.3) | 162 | (1.7) | 98 | (4.7) |
|  | 5+ visits |  | 112 | (0.0) | 19 | (0.0) | 31 | (0.1) | 36 | (0.4) | 26 | (1.2) |
| **Fragmentation indices** | | | | | | | | | | | | |
|  | Usual Provider of Care Index (UPC) | Mean (SD) | 0.8407 | (0.1740) | 0.8530 | (0.1719) | 0.8113 | (0.1749) | 0.7718 | (0.7437) | 0.7524 | (0.1683) |
|  | Continuity Of Care Index (COCI) | Mean (SD) | 0.7596 | (0.2399) | 0.7736 | (0.2441) | 0.7271 | (0.2232) | 0.6767 | (0.6521) | 0.6544 | (0.1951) |
|  | Sequential Continuity Index (SECON) | Mean (SD) | 0.8105 | (0.2126) | 0.8222 | (0.2158) | 0.7837 | (0.2000) | 0.7403 | (0.7134) | 0.7179 | (0.1771) |

**eTable 4. Baseline healthcare utilization and healthcare fragmentation of study participants aged 65+ years in South Korea, stratified by frailty status**

|  | | | *Total* (N= 138,135) | | *Fit (N=* 51,179*)* | | *Mild frailty (N=* 51,157*)* | | *Moderate frailty (N=* 24,055*)* | | *Severe frailty (N=* 11,744*)* | |
| --- | --- | --- | --- | --- | --- | --- | --- | --- | --- | --- | --- | --- |
|  | | | *n* | *(%)* | *n* | *(%)* | *n* | *(%)* | *n* | *(%)* | *n* | *(%)* |
| **Outpatient visit** | | | | | | | | | | | | |
|  | Number of visits | Mean (SD) | 31.0 | (29.7) | 17.6 | (15.9) | 30.2 | (24.3) | 44.8 | (33.4) | 65.0 | (45.5) |
|  | 10+ visits |  | 117812 | (85.3) | 35513 | (69.4) | 47251 | (92.4) | 23425 | (97.4) | 11623 | (99.0) |
|  | 20+ visits |  | 79448 | (57.5) | 15468 | (30.2) | 32671 | (63.9) | 20318 | (84.5) | 10991 | (93.6) |
|  | 30+ visits |  | 50685 | (36.7) | 6623 | (12.9) | 19031 | (37.2) | 15327 | (63.7) | 9704 | (82.6) |
|  | Involved providers | Mean (SD) | 1.9 | (0.7) | 1.7 | (0.7) | 1.9 | (0.7) | 2.1 | (0.7) | 2.3 | (0.6) |
|  | Provider transitions | Mean (SD) | 5.9 | (7.6) | 2.7 | (3.9) | 5.5 | (6.2) | 9.2 | (8.4) | 14.8 | (12.5) |
| **Hospitalization** | | | | | | | | | | | | |
|  | Number of hospitalizations | Mean (SD) | 0.6 | (1.7) | 0.2 | (0.9) | 0.5 | (1.5) | 1.0 | (2.1) | 1.9 | (2.9) |
|  | 1+ admissions |  | 35102 | (25.4) | 5232 | (10.2) | 12302 | (24) | 10226 | (42.5) | 7342 | (62.5) |
|  | 3+ admissions |  | 7795 | (5.6) | 611 | (1.2) | 1997 | (3.9) | 2393 | (9.9) | 2794 | (23.8) |
|  | 5+ admissions |  | 3603 | (2.6) | 327 | (0.6) | 946 | (1.8) | 1046 | (4.3) | 1284 | (10.9) |
|  | Hospital length of stay | Mean (SD) | 24.4 | (54.9) | 19.5 | (55.0) | 22.0 | (54.5) | 25.2 | (55.2) | 30.8 | (54.5) |
| **ER visit** | | | | | | | | | | | | |
|  | Number of ER visits | Mean (SD) | 0.1 | (0.5) | 0.0 | (0.2) | 0.1 | (0.4) | 0.2 | (0.6) | 0.4 | (1.0) |
|  | 1+ visits |  | 10349 | (7.5) | 1152 | (2.3) | 3139 | (6.1) | 3137 | (13) | 2921 | (24.9) |
|  | 3+ visits |  | 1006 | (0.7) | 43 | (0.1) | 155 | (0.3) | 294 | (1.2) | 514 | (4.4) |
|  | 5+ visits |  | 216 | (0.2) | 8 | (0.0) | 32 | (0.1) | 46 | (0.2) | 130 | (1.1) |
| **Fragmentation indices** | | | | | | | | | | | | |
|  | Usual Provider of Care Index (UPC) | Mean (SD) | 0.8320 | (0.1681) | 0.8612 | (0.1658) | 0.8290 | (0.1683) | 0.8011 | (0.1646) | 0.7813 | (0.1595) |
|  | Continuity Of Care Index (COCI) | Mean (SD) | 0.7532 | (0.2196) | 0.7899 | (0.2290) | 0.7509 | (0.2167) | 0.7127 | (0.2034) | 0.6860 | (0.1891) |
|  | Sequential Continuity Index (SECON) | Mean (SD) | 0.7904 | (0.2065) | 0.8263 | (0.2097) | 0.7857 | (0.2075) | 0.7526 | (0.1957) | 0.7314 | (0.1787) |

**eTable 5. Hazard ratio of all-cause mortality among study participants aged 45-64 years in Taiwan by fitting Cox regression model, stratified by frailty status**

|  |  | Total | |  | | Fit | | | Mild | | | | Moderate | | | | Severe | | | |  |
| --- | --- | --- | --- | --- | --- | --- | --- | --- | --- | --- | --- | --- | --- | --- | --- | --- | --- | --- | --- | --- | --- |
|  |  | aHR | (95% CI) | |  | | aHR | (95% CI) |  | aHR | | (95% CI) |  | aHR | | (95% CI) |  | aHR | | (95% CI) |  |
| Hospital trajectories (Number of hospitalizations) | | | |  | | | | |  | |  | |  | |  | |  | |  | |  |
|  | 0 | 1 |  | |  | | 1 |  |  | 1 | |  |  | 1 | |  |  | 1 | |  |  |
|  | 1 | 3.85 | (3.54-4.19) | |  | | 3.80 | (3.37-4.29) |  | 2.61 | | (2.25-3.03) |  | 2.57 | | (1.92-3.43) |  | 2.26 | | (1.25-4.10) |  |
|  | 2 | 10.33 | (9.24-11.55) | |  | | 12.41 | (10.41-14.80) |  | 6.45 | | (5.35-7.78) |  | 4.78 | | (3.46-6.60) |  | 3.03 | | (1.63-5.62) |  |
|  | 3+ | 32.47 | (29.87-35.29) | |  | | 47.42 | (41.87-53.71) |  | 19.10 | | (16.40-22.26) |  | 11.52 | | (8.72-15.23) |  | 8.44 | | (4.96-14.35) |  |
| ER trajectories (Number of ER visits) | | | | |  | |  |  |  |  | |  |  |  | |  |  |  | |  |  |
|  | 0 | 1 |  | |  | | 1 |  |  | 1 | |  |  | 1 | |  |  | 1 | |  |  |
|  | 1 | 2.07 | (1.91-2.25) | |  | | 1.77 | (1.58-1.99) |  | 1.84 | | (1.59-2.13) |  | 1.73 | | (1.31-2.27) |  | 2.01 | | (1.22-3.32) |  |
|  | 2 | 4.53 | (4.07-5.04) | |  | | 4.32 | (3.71-5.05) |  | 3.31 | | (2.75-3.98) |  | 2.61 | | (1.92-3.56) |  | 1.66 | | (0.90-3.04) |  |
|  | 3+ | 10.21 | (9.29-11.23) | |  | | 9.39 | (7.93-11.12) |  | 5.98 | | (5.06-7.08) |  | 4.34 | | (3.34-5.63) |  | 4.03 | | (2.62-6.20) |  |
| Usual Provider of Care Index (UPC) | | | |  | | | | |  | |  | |  | |  | |  | |  | |  |
|  | 1 | 1 |  | |  | | 1 |  |  | 1 | |  |  | 1 | |  |  | 1 | |  |  |
|  | 0.8-1 | 1.50 | (1.37-1.64) | |  | | 1.30 | (1.17-1.46) |  | 1.22 | | (1.02-1.46) |  | 0.68 | | (0.47-0.99) |  | 0.82 | | (0.35-1.93) |  |
|  | 0.6-0.8 | 1.49 | (1.37-1.62) | |  | | 1.22 | (1.10-1.36) |  | 1.21 | | (1.02-1.43) |  | 0.72 | | (0.51-1.03) |  | 1.04 | | (0.45-2.39) |  |
|  | 0.4-0.6 | 1.61 | (1.46-1.77) | |  | | 1.22 | (1.08-1.39) |  | 1.30 | | (1.08-1.55) |  | 0.65 | | (0.45-0.93) |  | 0.88 | | (0.38-2.06) |  |
|  | 0.2-0.4 | 2.28 | (1.78-2.93) | |  | | 1.69 | (1.14-2.50) |  | 1.70 | | (1.12-2.58) |  | 0.67 | | (0.31-1.42) |  | 1.16 | | (0.39-3.45) |  |
| Continuity of Care Index (COCI) | | |  | |  | |  |  |  |  | |  |  |  | |  |  |  | |  |  |
|  | 1 | 1 |  | |  | | 1 |  |  | 1 | |  |  | 1 | |  |  | 1 | |  |  |
|  | 0.8-1 | 1.84 | (1.64-2.06) | |  | | 1.67 | (1.45-1.93) |  | 1.35 | | (1.09-1.68) |  | 0.70 | | (0.46-1.09) |  | 0.75 | | (0.29-1.91) |  |
|  | 0.6-0.8 | 1.44 | (1.31-1.59) | |  | | 1.21 | (1.07-1.36) |  | 1.18 | | (0.98-1.43) |  | 0.67 | | (0.45-0.98) |  | 0.88 | | (0.38-2.08) |  |
|  | 0.4-0.6 | 1.52 | (1.40-1.65) | |  | | 1.23 | (1.11-1.36) |  | 1.18 | | (1.00-1.39) |  | 0.70 | | (0.50-0.99) |  | 0.97 | | (0.42-2.24) |  |
|  | 0.2-0.4 | 1.51 | (1.35-1.69) | |  | | 1.12 | (0.96-1.30) |  | 1.51 | | (1.22-1.86) |  | 0.64 | | (0.42-0.98) |  | 1.03 | | (0.43-2.46) |  |
|  | 0-0.2 | 0.60 | (0.29-1.27) | |  | | 0.71 | (0.34-1.50) |  | - | |  |  | - | |  |  | - | |  |  |
| Sequential Continuity Index (SECON) | | | | |  | |  |  |  |  | |  |  |  | |  |  |  | |  |  |
|  | 1 | 1 |  | |  | | 1 |  |  | 1 | |  |  | 1 | |  |  | 1 | |  |  |
|  | 0.8-1 | 1.77 | (1.62-1.94) | |  | | 1.59 | (1.42-1.78) |  | 1.40 | | (1.16-1.68) |  | 0.80 | | (0.54-1.17) |  | 0.78 | | (0.32-1.87) |  |
|  | 0.6-0.8 | 1.40 | (1.28-1.52) | |  | | 1.17 | (1.05-1.31) |  | 1.11 | | (0.93-1.33) |  | 0.60 | | (0.42-0.86) |  | 1.00 | | (0.43-2.31) |  |
|  | 0.4-0.6 | 1.50 | (1.36-1.64) | |  | | 1.11 | (0.98-1.26) |  | 1.17 | | (0.98-1.40) |  | 0.68 | | (0.47-0.97) |  | 0.94 | | (0.41-2.18) |  |
|  | 0.2-0.4 | 1.68 | (1.47-1.91) | |  | | 1.21 | (1.00-1.46) |  | 1.57 | | (1.25-1.97) |  | 0.86 | | (0.54-1.35) |  | 0.88 | | (0.33-2.34) |  |
|  | 0-0.2 | 1.09 | (0.79-1.50) | |  | | 0.85 | (0.56-1.29) |  | 1.88 | | (1.07-3.30) |  | 0.43 | | (0.06-3.14) |  | 3.78 | | (0.75-18.94) |  |

*Adjust for age, sex

**eTable 6. Hazard ratio of all-cause mortality among study participants aged 65+ years in Taiwan by fitting Cox regression model, stratified by frailty status**

|  |  | Total | |  | | Fit | | | Mild | | | | Moderate | | | | Severe | | | |
| --- | --- | --- | --- | --- | --- | --- | --- | --- | --- | --- | --- | --- | --- | --- | --- | --- | --- | --- | --- | --- |
|  |  | aHR | (95% CI) | |  | | aHR | (95% CI) |  | aHR | | (95% CI) |  | aHR | | (95% CI) |  | aHR | | (95% CI) |
| Hospital trajectories (Number of hospitalizations) | | | |  | | | | |  | |  | |  | |  | |  | |  | |
|  | 0 | 1 |  | |  | | 1 |  |  | 1 | |  |  | 1 | |  |  | 1 | |  |
|  | 1 | 2.00 | (1.92-2.09) | |  | | 1.88 | (1.68-2.10) |  | 1.79 | | (1.68-1.92) |  | 1.77 | | (1.63-1.92) |  | 1.75 | | (1.54-2.00) |
|  | 2 | 3.25 | (3.07-3.44) | |  | | 4.08 | (3.38-4.94) |  | 3.29 | | (2.96-3.66) |  | 2.34 | | (2.10-2.60) |  | 2.81 | | (2.45-3.22) |
|  | 3+ | 6.26 | (5.94-6.60) | |  | | 13.33 | (11.08-16.04) |  | 6.91 | | (6.13-7.77) |  | 4.53 | | (4.06-5.06) |  | 4.86 | | (4.29-5.49) |
| ER trajectories (Number of ER visits) | | | | |  | |  |  |  |  | |  |  |  | |  |  |  | |  |
|  | 0 | 1 |  | |  | | 1 |  |  | 1 | |  |  | 1 | |  |  | 1 | |  |
|  | 1 | 1.55 | (1.49-1.62) | |  | | 1.50 | (1.38-1.64) |  | 1.39 | | (1.30-1.49) |  | 1.34 | | (1.23-1.47) |  | 1.31 | | (1.16-1.48) |
|  | 2 | 2.16 | (2.05-2.28) | |  | | 1.92 | (1.66-2.23) |  | 1.90 | | (1.73-2.09) |  | 1.75 | | (1.57-1.94) |  | 1.64 | | (1.44-1.87) |
|  | 3+ | 3.27 | (3.12-3.44) | |  | | 3.67 | (3.09-4.36) |  | 2.77 | | (2.51-3.06) |  | 2.35 | | (2.13-2.59) |  | 2.23 | | (2.00-2.48) |
| Usual Provider of Care Index (UPC) | | | |  | | | | |  | |  | |  | |  | |  | |  | |
|  | 1 | 1 |  | |  | | 1 |  |  | 1 | |  |  | 1 | |  |  | 1 | |  |
|  | 0.8-1 | 1.16 | (1.10-1.22) | |  | | 1.07 | (0.98-1.17) |  | 0.93 | | (0.86-1.01) |  | 0.90 | | (0.78-1.03) |  | 0.88 | | (0.72-1.07) |
|  | 0.6-0.8 | 1.17 | (1.12-1.23) | |  | | 1.03 | (0.95-1.12) |  | 0.90 | | (0.83-0.97) |  | 0.83 | | (0.73-0.94) |  | 0.95 | | (0.79-1.15) |
|  | 0.4-0.6 | 1.21 | (1.15-1.27) | |  | | 1.02 | (0.93-1.12) |  | 0.92 | | (0.84-0.99) |  | 0.83 | | (0.72-0.94) |  | 0.86 | | (0.71-1.04) |
|  | 0.2-0.4 | 1.08 | (0.94-1.25) | |  | | 0.79 | (0.52-1.20) |  | 0.75 | | (0.58-0.96) |  | 0.77 | | (0.59-1.02) |  | 0.68 | | (0.50-0.94) |
| Continuity of Care Index (COCI) | | |  | |  | |  |  |  |  | |  |  |  | |  |  |  | |  |
|  | 1 | 1 |  | |  | | 1 |  |  | 1 | |  |  | 1 | |  |  | 1 | |  |
|  | 0.8-1 | 1.20 | (1.12-1.27) | |  | | 1.11 | (0.99-1.25) |  | 0.99 | | (0.89-1.09) |  | 0.88 | | (0.75-1.03) |  | 0.82 | | (0.65-1.02) |
|  | 0.6-0.8 | 1.16 | (1.10-1.22) | |  | | 1.06 | (0.97-1.17) |  | 0.90 | | (0.83-0.98) |  | 0.83 | | (0.72-0.95) |  | 0.95 | | (0.78-1.16) |
|  | 0.4-0.6 | 1.20 | (1.15-1.26) | |  | | 1.04 | (0.96-1.13) |  | 0.90 | | (0.84-0.97) |  | 0.87 | | (0.77-0.98) |  | 0.91 | | (0.75-1.09) |
|  | 0.2-0.4 | 1.14 | (1.07-1.21) | |  | | 0.95 | (0.84-1.07) |  | 0.87 | | (0.79-0.97) |  | 0.77 | | (0.67-0.90) |  | 0.82 | | (0.67-1.01) |
|  | 0-0.2 | 0.49 | (0.24-0.97) | |  | | 0.44 | (0.18-1.05) |  | 2.89 | | (0.72-11.57) |  | - | |  |  | 1.69 | | (0.24-12.11) |
| Sequential Continuity Index (SECON) | | | | |  | |  |  |  |  | |  |  |  | |  |  |  | |  |
|  | 1 | 1 |  | |  | | 1 |  |  | 1 | |  |  | 1 | |  |  | 1 | |  |
|  | 0.8-1 | 1.19 | (1.13-1.26) | |  | | 1.11 | (1.01-1.22) |  | 1.01 | | (0.92-1.10) |  | 0.88 | | (0.76-1.02) |  | 0.85 | | (0.69-1.04) |
|  | 0.6-0.8 | 1.16 | (1.10-1.21) | |  | | 1.02 | (0.94-1.12) |  | 0.89 | | (0.82-0.96) |  | 0.83 | | (0.73-0.95) |  | 0.88 | | (0.73-1.07) |
|  | 0.4-0.6 | 1.17 | (1.11-1.22) | |  | | 0.99 | (0.90-1.09) |  | 0.86 | | (0.80-0.94) |  | 0.79 | | (0.70-0.90) |  | 0.87 | | (0.72-1.06) |
|  | 0.2-0.4 | 1.27 | (1.20-1.36) | |  | | 1.07 | (0.94-1.23) |  | 0.93 | | (0.83-1.03) |  | 0.95 | | (0.81-1.10) |  | 1.03 | | (0.83-1.27) |
|  | 0-0.2 | 1.19 | (1.02-1.40) | |  | | 1.06 | (0.79-1.42) |  | 0.99 | | (0.77-1.26) |  | 1.30 | | (0.93-1.81) |  | 0.89 | | (0.52-1.53) |

**eTable 7. Hazard ratio of all-cause mortality among study participants aged 45-64 years in South Korea by fitting Cox regression model, stratified by frailty status**

|  |  | Total | |  | | Fit | | Mild | | | | Moderate | | | | Severe | | | |  |
| --- | --- | --- | --- | --- | --- | --- | --- | --- | --- | --- | --- | --- | --- | --- | --- | --- | --- | --- | --- | --- |
|  |  | aHR | (95% CI) | |  | aHR | (95% CI) |  | aHR | | (95% CI) |  | aHR | | (95% CI) |  | aHR | | (95% CI) |  |
| Hospital trajectories (Number of hospitalizations) | | | |  | | | |  | |  | |  | |  | |  | |  | |  |
|  | 0 | 1 |  | |  | 1 |  |  | 1 | |  |  | 1 | |  |  | 1 | |  |  |
|  | 1 | 1.94 | (1.72-2.18) | |  | 1.73 | (1.46-2.06) |  | 1.55 | | (1.26-1.89) |  | 1.79 | | (1.25-2.58) |  | 1.74 | | (0.88-3.46) |  |
|  | 2 | 3.93 | (3.37-4.58) | |  | 5.11 | (4.08-6.40) |  | 2.63 | | (2.02-3.43) |  | 2.28 | | (1.48-3.54) |  | 0.98 | | (0.40-2.40) |  |
|  | 3+ | 18.05 | (16.47-19.79) | |  | 18.86 | (16.10-22.09) |  | 13.77 | | (11.69-16.21) |  | 2.28 | | (1.48-3.54) |  | 7.19 | | (4.17-12.40) |  |
| ER trajectories (Number of ER visits) | | | | |  |  |  |  |  | |  |  |  | |  |  |  | |  |  |
|  | 0 | 1 |  | |  | 1 |  |  | 1 | |  |  | 1 | |  |  | 1 | |  |  |
|  | 1 | 4.61 | (4.09-5.19) | |  | 4.79 | (3.94-5.83) |  | 3.00 | | (2.44-3.68) |  | 2.76 | | (2.05-3.71) |  | 2.75 | | (1.80-4.20) |  |
|  | 2 | 11.42 | (9.50-13.72) | |  | 11.30 | (7.66-16.67) |  | 6.30 | | (4.54-8.75) |  | 7.41 | | (5.19-10.59) |  | 4.84 | | (2.90-8.09) |  |
|  | 3+ | 29.91 | (25.04-35.73) | |  | 27.26 | (16.89-44.02) |  | 6.30 | | (4.54-8.76) |  | 14.72 | | (10.49-20.66) |  | 7.09 | | (4.38-11.47) |  |
| Usual Provider of Care Index (UPC) | | | |  | | | |  | |  | |  | |  | |  | |  | |  |
|  | 1 | 1 |  | |  | 1 |  |  | 1 | |  |  | 1 | |  |  | 1 | |  |  |
|  | 0.8-1 | 1.57 | (1.41-1.74) | |  | 1.22 | (1.06-1.41) |  | 1.48 | | (1.23-1.80) |  | 0.97 | | (0.67-1.40) |  | 0.86 | | (0.44-1.66) |  |
|  | 0.6-0.8 | 1.58 | (1.42-1.75) | |  | 1.26 | (1.10-1.45) |  | 1.37 | | (1.123-1.67) |  | 1.14 | | (0.79-1.64) |  | 1.05 | | (0.55-2.02) |  |
|  | 0.4-0.6 | 1.7 | (1.51-1.93) | |  | 1.46 | (1.23-1.73) |  | 1.19 | | (0.94-1.51) |  | 1.23 | | (0.83-1.82) |  | 0.97 | | (0.48-1.93) |  |
|  | 0.2-0.4 | 1.62 | (0.93-2.80) | |  | 0.71 | (0.23-2.2) |  | 1.58 | | (0.65-3.84) |  | 1.69 | | (0.61-4.73) |  | 0.66 | | (0.08-5.10) |  |
| Continuity of Care Index (COCI) | | |  | |  |  |  |  |  | |  |  |  | |  |  |  | |  |  |
|  | 1 | 1 |  | |  | 1 |  |  | 1 | |  |  | 1 | |  |  | 1 | |  |  |
|  | 0.8-1 | 1.73 | (1.53-1.97) | |  | 1.41 | (1.17-1.69) |  | 1.51 | | (1.20-1.89) |  | 0.86 | | (0.55-1.33) |  | 0.93 | | (0.45-1.90) |  |
|  | 0.6-0.8 | 1.49 | (1.33-1.67) | |  | 1.14 | (0.97-1.33) |  | 1.43 | | (1.16-1.76) |  | 0.98 | | (0.66-1.45) |  | 0.80 | | (0.40-1.60) |  |
|  | 0.4-0.6 | 1.69 | (1.53-1.86) | |  | 1.35 | (1.18-1.54) |  | 1.33 | | (1.10-1.61) |  | 1.26 | | (0.89-1.80) |  | 1.15 | | (0.61-2.18) |  |
|  | 0.2-0.4 | 1.37 | (1.16-1.61) | |  | 1.25 | (1.01-1.55) |  | 1.16 | | (0.82-1.63) |  | 1.23 | | (0.7452.02) |  | 0.46 | | (0.16-1.34) |  |
|  | 0-0.2 | 0.93 | (0.35-2.48) | |  | 1.11 | (0.41-2.97) |  | - | |  |  | - | |  |  | - | |  |  |
| Sequential Continuity Index (SECON) | | | | |  |  |  |  |  | |  |  |  | |  |  |  | |  |  |
|  | 1 | 1 |  | |  | 1 |  |  | 1 | |  |  | 1 | |  |  | 1 | |  |  |
|  | 0.8-1 | 1.81 | (1.63-2.01) | |  | 1.46 | (1.26-1.68) |  | 1.63 | | (1.35-1.98) |  | 1.08 | | (0.74-1.58) |  | 1.15 | | (0.59-2.23) |  |
|  | 0.6-0.8 | 1.52 | (1.37-1.68) | |  | 1.21 | (1.05-1.40) |  | 1.32 | | (1.08-1.61) |  | 1.03 | | (0.72-1.49) |  | 0.81 | | (0.42-1.56) |  |
|  | 0.4-0.6 | 1.53 | (1.35-1.74) | |  | 1.20 | (1.02-1.43) |  | 1.13 | | (0.89-1.44) |  | 1.22 | | (0.82-1.79) |  | 1.02 | | (0.52-2.02) |  |
|  | 0.2-0.4 | 1.33 | (1.07-1.66) | |  | 1.04 | (0.76-1.43) |  | 1.29 | | (0.88-1.89) |  | 1.25 | | (0.68-2.29) |  | 0.68 | | (0.19-2.43) |  |
|  | 0-0.2 | 1.08 | (0.68-1.70) | |  | 1.31 | (0.82-1.82) |  | 0.63 | | (0.16-2.53) |  | - | |  |  | - | |  |  |

*Adjust for age, sex

**eTable 8. Hazard ratio of all-cause mortality among study participants aged 65+ years in South Korea by fitting Cox regression model, stratified by frailty status**

|  |  | Total | |  | | Fit | | Mild | | | | Moderate | | | | Severe | | | |  |
| --- | --- | --- | --- | --- | --- | --- | --- | --- | --- | --- | --- | --- | --- | --- | --- | --- | --- | --- | --- | --- |
|  |  | aHR | (95% CI) | |  | aHR | (95% CI) |  | aHR | | (95% CI) |  | aHR | | (95% CI) |  | aHR | | (95% CI) |  |
| Hospital trajectories (Number of hospitalizations) | | | |  | | | |  | |  | |  | |  | |  | |  | |  |
|  | 0 | 1 |  | |  | 1 |  |  | 1 | |  |  | 1 | |  |  | 1 | |  |  |
|  | 1 | 1.54 | (1.46-1.62) | |  | 1.61 | (1.42-1.83) |  | 1.53 | | (1.41-1.67) |  | 1.33 | | (1.20-1.48) |  | 1.45 | | (1.26-1.67) |  |
|  | 2 | 2.06 | (1.92-2.20) | |  | 2.07 | (1.67-2.56) |  | 1.99 | | (1.77-2.24) |  | 1.82 | | (1.60-2.06) |  | 1.90 | | (1.63-2.22) |  |
|  | 3+ | 4.64 | (4.42-4.87) | |  | 6.67 | (5.77-7.71) |  | 4.67 | | (4.26-5.12) |  | 4.08 | | (3.70-4.51) |  | 3.84 | | (3.41-4.33) |  |
| ER trajectories (Number of ER visits) | | | | |  |  |  |  |  | |  |  |  | |  |  |  | |  |  |
|  | 0 | 1 |  | |  | 1 |  |  | 1 | |  |  | 1 | |  |  | 1 | |  |  |
|  | 1 | 2.10 | (1.98-2.22) | |  | 2.48 | (2.09-2.93) |  | 2.20 | | (1.99-2.43) |  | 2.20 | | (1.98-2.43) |  | 1.57 | | (1.39-1.76) |  |
|  | 2 | 3.47 | (3.16-3.8) | |  | 3.23 | (2.12-4.91) |  | 3.27 | | (2.68-3.98) |  | 3.27 | | (2.68-3.98) |  | 2.41 | | (2.06-2.81) |  |
|  | 3+ | 5.60 | (5.08-6.18) | |  | 3.57 | (1.78-7.15) |  | 5.04 | | (3.84-6.61) |  | 5.04 | | (3.84-6.61) |  | 4.25 | | (3.69-4.89) |  |
| Usual Provider of Care Index (UPC) | | | |  | | | |  | |  | |  | |  | |  | |  | |  |
|  | 1 | 1 |  | |  | 1 |  |  | 1 | |  |  | 1 | |  |  | 1 | |  |  |
|  | 0.8-1 | 1.11 | (1.05-1.16) | |  | 1.16 | (1.05-1.27) |  | 0.93 | | (0.85-1.01) |  | 0.87 | | (0.77-0.97) |  | 0.81 | | (0.69-0.96) |  |
|  | 0.6-0.8 | 1.35 | (1.29-1.42) | |  | 1.27 | (1.15-1.40) |  | 1.21 | | (1.12-1.32) |  | 1.11 | | (0.99-1.25) |  | 0.94 | | (0.80-1.11) |  |
|  | 0.4-0.6 | 1.47 | (1.30-1.56) | |  | 1.35 | (1.19-1.54) |  | 1.32 | | (1.20-1.46) |  | 1.17 | | (1.02-1.33) |  | 1.08 | | (0.90-1.29) |  |
|  | 0.2-0.4 | 1.84 | (1.41-2.39) | |  | 1.86 | (1.05-3.28) |  | 1.71 | | (1.07-2.72) |  | 1.01 | | (0.5671.79) |  | 1.77 | | (1.03-3.05) |  |
| Continuity of Care Index (COCI) | | |  | |  |  |  |  |  | |  |  |  | |  |  |  | |  |  |
|  | 1 | 1 |  | |  | 1 |  |  | 1 | |  |  | 1 | |  |  | 1 | |  |  |
|  | 0.8-1 | 1.04 | (0.97-1.10) | |  | 1.04 | (0.92-1.18) |  | 0.89 | | (0.81-0.99) |  | 0.79 | | (0.69-0.91) |  | 0.76 | | (0.63-0.90) |  |
|  | 0.6-0.8 | 1.22 | (1.16-1.29) | |  | 1.26 | (1.14-1.41) |  | 1.03 | | (0.94-1.13) |  | 0.96 | | (0.85-1.09) |  | 0.85 | | (0.72-1.01) |  |
|  | 0.4-0.6 | 1.41 | (1.34-1.48) | |  | 1.36 | (1.24-1.50) |  | 1.25 | | (1.15-1.35) |  | 1.14 | | (1.02-1.28) |  | 1.01 | | (0.86-1.19) |  |
|  | 0.2-0.4 | 1.45 | (1.33-1.58) | |  | 1.04 | (0.86-1.26) |  | 1.39 | | (1.19-1.61) |  | 1.29 | | (1.07-1.55) |  | 1.24 | | (0.99-1.55) |  |
|  | 0-0.2 | 1.45 | (0.65-3.23) | |  | 1.95 | (0.81-4.70) |  | - | |  |  | 3.27 | | (0.46-23.27) |  | - | |  |  |
| Sequential Continuity Index (SECON) | | | | |  |  |  |  |  | |  |  |  | |  |  |  | |  |  |
|  | 1 | 1 |  | |  | 1 |  |  | 1 | |  |  | 1 | |  |  | 1 | |  |  |
|  | 0.8-1 | 1.13 | (1.07-1.19) | |  | 1.13 | (1.02-1.25) |  | 0.99 | | (0.90-1.08) |  | 0.89 | | (0.79-1.00) |  | 0.82 | | (0.69-0.96) |  |
|  | 0.6-0.8 | 1.29 | (1.22-1.36) | |  | 1.27 | (1.15-1.40) |  | 1.13 | | (1.04-1.23) |  | 1.03 | | (0.92-1.16) |  | 0.86 | | (0.73-1.01) |  |
|  | 0.4-0.6 | 1.35 | (1.28-1.43) | |  | 1.27 | (1.12-1.43) |  | 1.15 | | (1.04-1.27) |  | 1.04 | | (0.92-1.19) |  | 1.05 | | (0.88-1.24) |  |
|  | 0.2-0.4 | 1.52 | (1.39-1.66) | |  | 1.45 | (1.20-1.73) |  | 1.31 | | (1.13-1.51) |  | 1.38 | | (1.15-1.66) |  | 1.27 | | (0.99-1.63) |  |
|  | 0-0.2 | 1.49 | (1.23-1.82) | |  | 1.49 | (1.06-2.09) |  | 1.41 | | (1.02-1.96) |  | 1.27 | | (0.83-1.93) |  | 2.48 | | (1.27-4.83) |  |

*Adjust for age, sex
